# Comparative analysis of multiple deep learning models with mitigation-driven approaches for enhanced Alzheimer’s disease classification

**DOI:** 10.1038/s41598-025-27914-w

**Published:** 2025-11-22

**Authors:** Areej Y. Bayahya, Haneen Banjar, Omar Talabay, Sultan H. Alamri

**Affiliations:** 1 Computer Science Department, Faculty of Computing and Information Technology, King Abdulaziz University, 21589 Jeddah, Saudi Arabia; 2 Software Engineering Department, College of Engineering, University of Business and Technology (UBT), 21448 Jeddah, Saudi Arabia; 3 Center of Research Excellence in Artificial Intelligence and Data Science, King Abdulaziz University, 21589 Jeddah, Saudi Arabia; 4 Institute of Genomic Medicine Sciences, King Abdulaziz University, 21589 Jeddah, Saudi Arabia; 5 Centre of Artificial Intelligence in Precision Medicines, King Abdulaziz University, 21589 Jeddah, Saudi Arabia; 6 Future Artificial Intelligence Company (Humain), 13511 Riyadh, Saudi Arabia; 7 Department of Family Medicine, Faculty of Medicine, King Abdulaziz University, 21589 Jeddah, Saudi Arabia

**Keywords:** AI, Dementia, Deep Neural Network, Caps Network, ViT, CNNs, Cognitive ageing, Medical research

## Abstract

Alzheimer’s disease diagnosis from structural MRI remains challenging in clinical practice. While deep learning shows promise for automated dementia detection, comprehensive comparisons of different neural network approaches are lacking. It analyzed T1-weighted MRI scans comprised 14,983 2D grid images derived from 1346 unique patients. Ten coronal brain slices spaced 2mm apart were arranged in 512 × 512-pixel grids using our 2D coronal-10 slicing sMRI methodology to preserve anatomical relationships while reducing computational demands. Ten deep learning architectures were systematically compared, including traditional CNNs, Vision Transformers, and Capsule Networks. Patient-level data splitting prevented information leakage. ECAResNet269 achieved the highest balanced accuracy (63%), with mild performance across all classes: dementia (38% sensitivity/77% specificity), MCI (72% sensitivity/66% specificity), and healthy controls (44% sensitivity/90% specificity). Class imbalance mitigation strategies substantially improved model performance, with combined SMOTE, cost-sensitive learning, and focal loss approaches achieving 74% balanced accuracy and (78% CN, 76%MCI, 69% AD) sensitivity in the ECAResNet269 model. Pretrained CNNs architectures substantially outperformed advanced methods–Vision Transformer and CapsNets showed complete classification failure. The 2D grid method retained 96% of diagnostic information compared to 3D approaches while providing 4.2 × faster processing. Traditional CNNs architectures remain most effective for medical neuroimaging classification. ECAResNet269 achieved clinically relevant performance suitable for dementia screening applications. The 2D grid methodology successfully balances diagnostic accuracy with computational efficiency, enabling deployment on standard clinical hardware.

## Introduction

Multiple deep neural network architectures have fundamentally transformed the domain of medical imaging, presenting unprecedented capabilities for the classification in Magnetic Resonance Imaging (MRI)^1^. In the context of neuroimaging, the identification of structural anomalies, particularly those associated with conditions such as Mild Cognitive Impairment (MCI), requires the employment of models that can handle the intricate,

overlapping, and frequently subtle distinctions in brain structures^2^.

Despite the prevalent dominance of traditional model such as convolutional neural networks (CNNs) and their variations in medical image investigation filed, a notable insight is that the specific architectural design is not the primary factor in attaining optimal performance outcomes. For instance, in challenges like MCI, numerous researchers utilize the same architectural framework but obtain different outcomes. A frequently disregarded consideration that domain-specific expertise is related to the task at hand can provide significant advantages beyond simply augmenting the number of layers within a CNN^3^. Despite that CNNs is better at capturing local spatial details acquisition owing to their hierarchical architecture and has ability to learn features autonomously, negating the necessity for prior domain knowledge or manual segmentation^3^. However, CNNs fall short due to the complexity of the data, the variability inherent in imaging protocols, and the necessity for elevated sensitivity and specificity^1^.

Recent advancements in artificial intelligence (AI) have introduced promising deep learning architectures, such like Vision Transformer (ViT) and Capsule Networks (CapsNets) for medical imaging classification. However, these models remain largely unexamined for dementia diagnosis using 2D-10 slices coronal sMRI. Coronal slicing preserves anatomical orientation critical for neurological assessment. Previous studies have not systematically evaluated modern architectures using this approach. CapsNets have not yet been implemented to sMRI-based dementia diagnosis. Although ViT have demonstrated across various medical imaging. However, it is conspicuous absence of studies that have applied them specifically to 2D-10 slices coronal sMRI-based dementia classification.

**Fig. 1:**
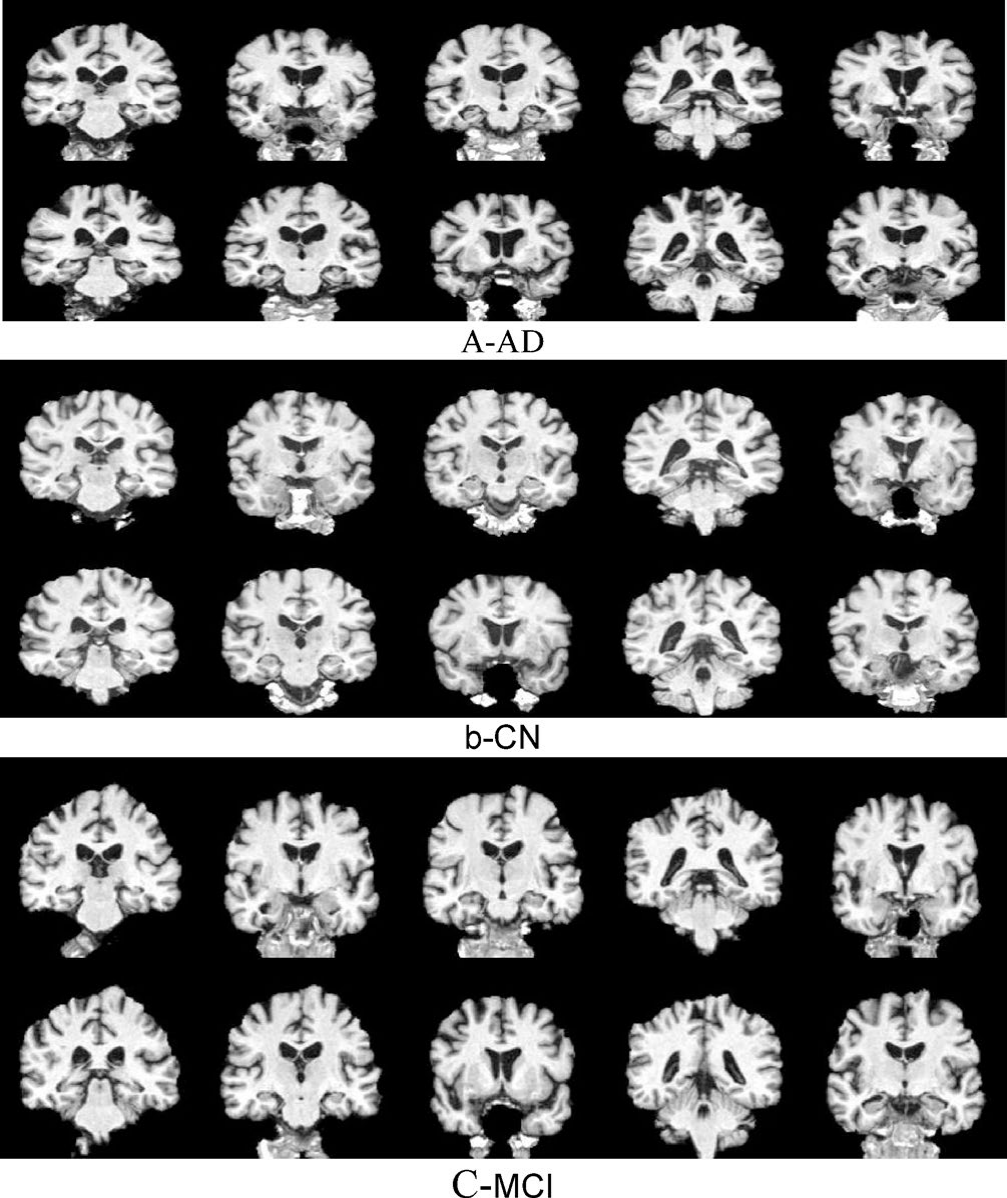
2D Grid Slices images of the Brain: (**a**) AD,(**b**) CN,(**C**) MCI.

**Fig. 2:**
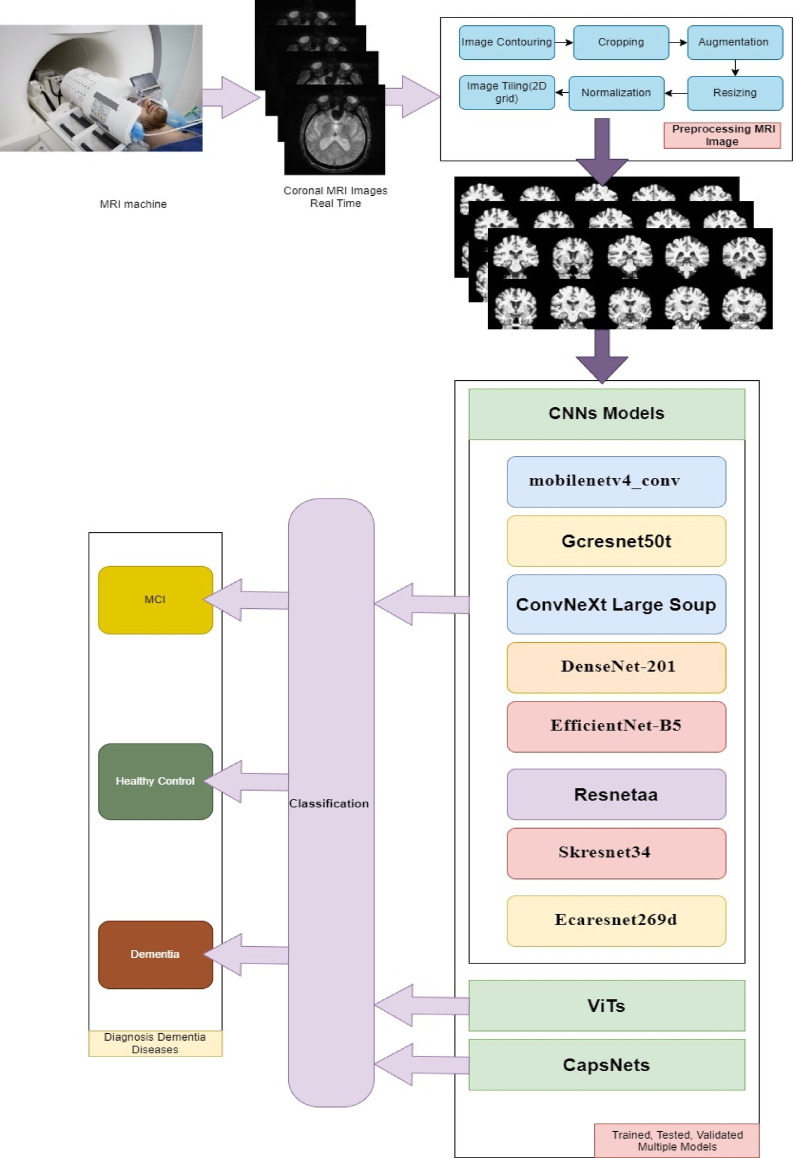
Framework of Multiple Model.

This study claims that classification of dementia using deep learning requires more integration of mitigation methods and preprocessing techniques, such as focusing on 2D Coronal or Regions of Interest (ROI) with advanced preprocessing techniques. Traditional batch processing architectures may not efficiently capture fine-grained patterns in medical imaging data, necessitating an improved approach for feature extraction and classification. This study investigates CapsNets and ViT to examine new possibilities for leveraging global context in brain imaging. Additionally, novel key in this study lies in applying 2D coronal with 10-slices methodology to compare CNNs, ViT, and CapsNet architectures with/without mitigation approaches as shown in Figs. 1 and 2. This approach maintains anatomical coherence while enabling computational efficiency. No prior work has systematically evaluated these architectures using 2D coronal-10 slicing sMRI as shown in Fig. 1. This study examined CNN models to evaluate and classify sMRI images for dementia patients. Based on high-performance criteria of pretrained CNN models, this study selected 8 out 20 models and elicited from a different family of CNN. This study focuses on evaluating the accuracy, F1, Precision, specificity and sensitivity of Dementia.

This study seeks to explore the following aims:

- To develop a multi-architecture deep learning framework that compares CNNs, ViT, and CapsNets for sMRI based on 2D coronal slices.
- To explore and compare multiple CNN families and select the best-performing pre-trained models from each family for image classification in dementia detection.
- To assess the impact of CNN families, ViT, and CapsNets on sMRI classification for demented and MCI patients
- To classify patients into MCI, healthy control and demented categories using CNN family models, ViT and CapsNets.
- To evaluate each model based on performance metrics, including accuracy, sensitivity, F1, Precision, specificity, and others for each model.
- To investigate whether these models can achieve high performance without applying additional computer vision techniques or argumentation factors.
- To address the limitations in traditional approaches by integrating more adaptive and efficient data handling and analysis techniques

This article presents the Literature Review, Methodology, Performance Evaluation and Discussion of Results, followed by the Conclusion and References. Each section provides a structured analysis, ensuring a comprehensive understanding of the research.

## Literature review

Modern breakthroughs in AI and deep learning technologies have been propelled by new neural network architectures. Among these, CNN have become fundamental in computer vision. It excels in object detection, image classification, and segmentation due to their capability for effective capturing spatial hierarchies in visual data. CNN’s hierarchical architecture provides automatic feature extraction, essential for handling high-dimensional inputs.

The core components of a CNN encompass four layers: convolutional, pooling, nonlinear activation, and fully connected layers. Preprocessing is usually applied to an image before it enters the network through the input layer. Subsequently, it is processed through a sequence of alternately arranged convolutional and pooling layers, culminating in classification using fully connected layers^4,5^.

Additionally, ViT and CapsNet are newly models represent a paradigm shift in visual recognition, challenging the dominance of CNNs. CapsNets offer an innovative strategy to address the short comings of CNNs. It preserves the spatial hierarchies and handles the viewpoint variations. It constitutes a type of deep neural network engineered to capture complex spatial hierarchies and interrelations within data. It enhances image recognition and segmentation. Unlike CNN, CapsNets use capsules as vectors that encapsulate attributes such as size, position, and orientation of entities within an image^4,5^. ViT is an advanced architecture dedicated for image classification. ViT architecture employs self-attention methods to capture global relationships across image patches^6^.It consists of an embedding layer, an encoder, and a classifier head. Then, the model processes input images by partitioning them into non-overlapping patches, where each patch treating as a token.

A lot of studies investigate MRI brain images scans in the identification of dementia. Dementia is delineated by severe symptoms such as memory impairment and cognitive decline It affects more than 47 million people worldwide with estimations anticipated 131 million by 2050, as reported in the World Alzheimer Report 2016.

The early diagnosis of MCI is crucial as it facilitates the recognition of those at risk for Alzheimer’s disease (AD) and lead to more effective treatment, thereby potentially postponing the progression of the disease. To address these issues , numerous investigations have employed deep neural networks such as^7–19^. Carcagnì et al.^7^ objected to improve the automated classification of dementia using MRI brain imaging data by evaluating DenseNet, EfficientNet and ResNet for associating images to clinical diagnoses. Experiments were executed with various benchmarks were established by altering the quantity of slices per subject derived from 3D voxels. The findings demonstrated that deeper ResNet and DenseNet architectures outperformed on performance in comparison to their less complex equivalents.

Recent developments in neural networks and deep learning technologies have significantly influenced the domains of medical imaging, healthcare data analytics, and clinical diagnostics. Herzog, N.J. and G.D. Magoulas^8^ concentrate on early diagnosis and progressive dementia through the utilization of MRI classification with transfer learning architectures. CNN serve as the foundational model, with fully connected layers employing either Support Vector Machines (SVMs) or SoftMax functions for the classification task. The prediction process is predicated on analysing brain asymmetry, which serves as a predictive indicator for early dementia. In experimental involving 300 simulations across a variety of MRI classification tasks, the CNN-based models utilizing SVM output layers outperformed those with SoftMax layers, particularly during shorter training intervals. Early dementia was diagnosed with an average accuracy of 90.25%, while the accuracy for diagnosing progressive dementia reached 95.90% using the SVM-enhanced architecture. Nonetheless, , the study’s limitations include the small dataset size (comprising 600 images), limited access to clinical data, and the prospective necessity for more powerful hardware for larger-scale applications^8^.

Furthermore, other study was developed a CNN architecture to detect distinct AD features from MRI images. It is focusing on four progressive stages of dementia. Murugan et al.^11^, generated a high-resolution disease probability maps model and precise visual representations of individual AD risk, despite the challenge of class imbalance in datasets such as those provided by Kaggle. The DEMentia NETwork (DEMNET) achieved impressive results, with 95.23% accuracy and an Area Under the Curve (AUC) of 97% on the Kaggle dataset, outperforming existing methods. The efficacy of the model was also evaluated utilizing the ADNI dataset. However, limitations include the model’s testing on only two datasets, the potential for enhanced efficacy through the application of alternative foundational models such as Inception or Residual Networks, and the suggestion that certain pre-processing procedures may be deemed unnecessary. The investigation also posits that with more data and computational resources, fine-tuning the pre-trained convolutional layers, potentially leading to further performance enhancement.

Moreover, enhanced diagnostic methodologies are crucial for the identification of cognitive impairments caused by various factors. Qiu, S., et al.^9^ introduced a deep learning framework that systematically diagnoses dementias using a combination of clinical data, neuropsychological assessments, neuroimaging modalities, and functional assessments such as CNN. The framework’s accuracy is favourably comparable to that of neurologists and neuroradiologists. Additionally, interpretability techniques that the model’s predictions align with specific patterns of brain degeneration and neuropathological findings. However, the models tend to default to an AD diagnosis in instances of mixed dementia, inadequately address atypical forms of AD, and lack the capability distinguish between different types of MCI. Furthermore, the dataset utilized on AD, which may restrict the ability to identify dementias that are not classified as AD. In additions, Luo, S., X. Li, and J. Li^10^ introduced an automated algorithm for the recognition of AD using neural deep learning on 3D brain MRI data. It utilized a CNN that composed of three clusters of processing layers, succeeded by two fully connected classification layers. The trained model and tested model applied on MRI data from the ADNI, which included scans from 47 individuals diagnosed with AD and 34 normal controls. The algorithm demonstrated high detection accuracy, achieving a sensitivity of 1.0 and a specificity of 0.93. However, limitations include the necessity for more efficient data processing methodologies and CNN architectures, lack of rotational invariance in the MRI data, and a small, imbalanced dataset.

Another investigation conducted by Torghabeh et al.^13^ proposes for the implementation of CNN architecture to classify AD severity utilizing MRI scans. The objective is to leverage pre-trained CNNs as a decision support mechanism for medical physicians, , facilitating the anticipation of dementia severity. The model was trained using the conventional Kaggle dataset, with class imbalance rectified using the Synthetic Minority Oversampling Technique (SMOTE). By fine-tuning the VGGNet16 with the ReduceLROnPlateau approach, the model achieved a98.61%accuracy and99%specificity for multiclass classification, thereby surpassing existing methodologies. Modulating the Initial Learning Rate (ILR) during training phase helped the model converge more effectively.

The prediction of the transition from MCI to AD dementia is challenging. MRI serves a crucial role in diagnosing MCI and AD by facilitating the examination of both brain structure and function. Gorji and Naima Kaabouch^12^ discuss MCI, as a transitional phase that exists between normative cognitive function and AD, a severe neurodegenerative disorder that predominantly affects individuals aged 65 and older. The investigation employed a deep learning approach, specifically a CNN, to accurately differentiate between cognitively healthy subjects and those exhibiting early and late stages of MCI (EMCI and LMCI) based on analysis of MRI scans. Utilizing data derived from 600 participants, the model achieved high accuracy rates 93%, particularly in the discrimination between CN individuals and those with LMCI. However, the study’s limitations include the small sample size and indicate the necessity for subsequent research and refinement of the CNN-based analytical framework.

Furthermore, Li, H., et al.^14^ provided CNN model utilizing MRI images obtained from a cohort of 2146 subjects to predict MCI progression in a time-to-event analysis. The concordance index of the model was 0.762 when tested with 439 subjects from ADNI and 0.781 when evaluated on 40 participants from the Australian Imaging Biomarkers and Lifestyle (AIBL) dataset. The integration of initial clinical metrics significantly enhanced the model’s efficacy, culminating in a C-index of 0.864. This methodological approach predicts AD progression that facilitate the enrolment process for clinical trials. However, the study was limited to the hippocampal region and initial datasets, with potential improvements expected from applying the method to whole-brain MRI data, incorporating longitudinal data, and addressing the continuum of AD progression.

Moreover, AD represents a widely prevalent neurodegenerative disorder, with its initial phase, MCI, characterized by subtle alterations in the subcortical structures of the temporal lobe. Timely identification through neuroimaging techniques such as brain MRI is of paramount importance yet presents considerable difficulties. Tomassini, S., et al.^15^ introduced CLAUDIA, a decision-support system based on Convolutional Long Short-Term Memory (ConvLSTM) architecture, designed for diagnosing AD from 3D MRI images. The system, which has been developed and tested with 438 scans from the ADNI-1 collection. CLAUDIA’s multiclass classifier outperforms binary classifiers in the identification of MCI and AD, thereby illustrating its effectiveness in a cloud-based, machine-independent operational framework.

Numerous investigations have examined the challenges of diagnosing dementia and AD. These challenges are attributable to neurodegeneration in the brain. In light of the current lack of effective treatments, the significance of early diagnostic measures is crucial to prevent disease progression. Mohammed, B.A., et al.^16^ conducted an evaluation of diverse AI algorithms using the OASIS dataset. Their analysis evaluated ResNet-50 and AlexNet. As well as hybrid methodologies that integrate ResNet-50 + SVM and AlexNet + SVM. The random forest algorithm attained the highest accuracy at 94%. The evaluation of the MRI dataset showed that while all models performed well, the hybrid approaches surpassed the purely deep learning models in effectiveness. Specifically, the hybrid model SVM + AlexNet achieved an accuracy of 94.8%, with elevated specificity, sensitivity, and AUC scores. The study’s limitation lies in the need additional research to substantiate these findings.

Pradhan, A., J. Gige, and M. Eliazer^18^ presented a model that integrates DenseNet169 and VGG19 architectures. It analysed MRI brain images and aimed to identifying both the presence and severity of AD. However, additional investigations are necessary to implement this model in clinical environments. It deployed practically on a web-based platform, and validate its efficacy on a larger dataset.

Moreover,Sai, P.A., et al.^17^ explored the application of CNN for early detection by utilizing AlexNet. It trained on specific datasets, to extract pertinent features for the classification. The study investigates the effectiveness of this methodology in enhancing the accuracy and speed of AD diagnosis.

Another investigation^19^ concentrated on the CNN-based VUNO Med-DeepBrain AD (DBAD). It compared and evaluated Alzheimer’s diagnosis accuracy with medical experts using MRI data. The study conducted by Kim, J.S., et al.^19^ attained an accuracy of 87.1%, sensitivity of 93.3% and specificity of 85.5%. Its slightly outperforming the performance of the medical experts, who had an accuracy of 84.3%, sensitivity of 80.0%,while maintaining the same specificity of 85.5%.This finding implies suggests.

DBAD could serve as a valuable tool for non-specialists in diagnosing AD. However, it is essential to acknowledge the necessity for further expert validation and the potential trade-offs in processing speed that may arise when enhancing the algorithm which have been recognized as limitations.

Despite the significant progress achieved in the application of CNNs, CapsNets, and ViTs for the analysis of MRIs images, prior investigations reveal inconsistencies in findings attributable to various methodological gaps. These include an absence of comprehensive explanations concerning data preprocessing techniques, a lack of transparency in the optimization of hyperparameters and fine-tuning strategies. As well as unclear data partitioning for training, validation, and testing, which affects generalization and reproducibility. In light of these identified deficiencies, this study aims to develop an advanced AI framework comparing CNNs, CapsNets, and ViTs within a well-structured preprocessing and evaluation pipeline that used 2D coronal slices. The proposed methodology optimized model tuning and rigorous validation, ultimately improving the reliability and reproducibility of AI-driven medical image classification and analysis.

## Methodology

AI-based computational approaches are presented in the multiple CNNs, ViT and CapsNets models as generalizable approach as shown in Fig. 2. This study proposes multiple models combining high-performance criteria of pretrained CNN, ViT and CapsNets models as shown in Fig. 2. In addition, this study applied Class imbalance mitigation strategies to improved model performance, with combined SMOTE, cost-sensitive learning, and focal loss approaches. Consequently, it conducted a comparative analysis of the sensitivity, accuracy, specificity, F1, receiver operating characteristic (Roc) curve, and others associated with the classification of medical imaging utilizing sMRI in the context of early-stage MCI.

This research primarily focused on pre-trained image classification models but can be extended the capability to be adapted for various other computer vision detection. It selected 2D coronal slicing to preserve anterior–posterior anatomical relationships as shown in Fig. 1. This orientation provides optimal feature representation for the evaluated architectures. This study employed Timm stands for PyTorch Image Models. This library, developed in Python that provides a large collection of pre-trained deep learning models tailored for computer vision applications, particularly in image classification. It is widely used for its efficiency, diversity of models, and its seamless integration into PyTorch environments. It provides in excess of 900 pre-trained models crossing a multitude of architectures. The models have been trained utilizing popular datasets such as ImageNet, CIFAR, among others. It Includes utilities designed for the fine-tuning of pre-trained models to accommodate custom datasets. Additionally, all experiments were conducted on NVIDIA A1000 GPUs (4GB VRAM) with Intel Xeon processors. We systematically profiled computational requirements across all ten architectures, measuring: (1) trainable parameters, (2) memory consumption during training and inference, (3) training time per epoch, and (4) inference time per sample. Memory profiling was performed using PyTorch’s built-in memory tracking, while timing measurements represent averages across 1000 iterations with early stopping to ensure statistical reliability. Due to the 4GB memory constraint, larger models required gradient accumulation and mixed-precision training to fit within available memory.

This study conducted on CapsNets, ViT and eight different family models of CNN which includes: ConvNeXt, DenseNet, EfficientNet, ECAResNet, MobileNetV4, GCResNet, ResNet-AA, skresnet34 as shown in Fig. 2 and Table 1. In the following the subsections for choosing the pretrained models and performance criteria, ViT implementation Strategy, and CapsNets architecture and dynamic routing implementation^20^.

### Pre-trained models

This study utilizes eight distinct pretrained models, which are briefly summarized in Table 1 below.

**Table 1:** Pre-trained models based high performance criteria.

Pretrained models:	Features	Library
ConvNeXt^21^	four stages: Stage 1 (96 channels, 3 blocks), Stage 2 (192 channels, 3 blocks), Stage 3 (384 channels, 27 blocks), and Stage 4 (768 channels, 3 blocks)	PyTorch Image Models (Timm) library
Densely Connected Convolutional Networks (DenseNets-201)^22^	Dense Blocks, Transition Layers, Growth Rate, Global Feature Concatenation. and Classifier. It has a 1 × 1 convolution to diminish the feature maps number and a 2 × 2 average pooling layer to down-sample spatial dimensions	
EfficientNet -B5^23,24^	The number of channels progressively increasing: 32, 40, 64, 112, 192, 320 respectively form (Stage 2) to (Stage 7). The final stage employs a 1 × 1 convolution to consolidate features into a compact representation. A global average pooling layer follows, reducing the spatial dimensions into a single feature vector	
Skresnet34.ra_in1k model^25^	It enhances the traditional ResNet-34 framework by integrating Selective Kernel (SK) units	
Ecaresnet269d.ra2_in1k^26–28^	It has a three-layer configuration. Each layer using a 3 × 3 convolution and replacing the original single 7 × 7 convolution	
Mobilenetv4_conv_aa_large^29^	It has the Universal Inverted Bottleneck (UIB) including ConvNext, Feed Forward Network (FFN), Inverted Bottleneck (IB), and a novel Extra Depth wise (ExtraDW) variant	
Gcresnet50t.ra2_in1k^30^	It is the integration of Global Context (GC) attention, modified tiered stem, and residual bottleneck blocks	
Resnetaa101d.sw_in12k_ft_in1k^28,31,32^	It has a three-layer stem that integrates 3 × 3 convolutions with pooling	

### Performance criteria

This study utilities designed for the fine-tuning of pre-trained models to accommodate custom datasets. In the following the performance criteria steps for electing the models.

Step 1: Define Performance Criteria.

Before selecting the optimal model, it is clarifying the criteria for assessing “optimal performance”. Common criteria might include:

- Top-1 Accuracy (Higher is better)—The percentage of correctly classified instances based on the top-1 predicted label.
- Top-5 Accuracy (Higher is better)—The percentage of correctly classified instances based on the top-5 predicted label.
- Error Rate (Lower is better)—for both Top-1 and Top-5 evaluations.
- Parameter Count—Models with fewer parameters are more computationally efficient.
- Performance Score (Higher is better)—An overarching metric that combining various performance factors.
- Normalized Scores (norm_top1, norm_top5)—Utilize normalized values for comparison if they are provided.

Step 2: Filter Key Attributes.

Concentrate on important columns which are top1, top5, param_count, performance score.

Step 3: Normalize Data.

Normalize all criteria to a uniform scale (0–1) to make more straightforward comparisons.

Step 4: Rank Models.

Assess and rank models based on prioritized criteria which are : Primary Focus where use performance score or top1 accuracy as the deciding determinant. Tie-Breakers: Use top5 accuracy and lower param_count for enhanced efficiency.

Step 5: Evaluate Trade-Offs.

A model with the highest performance may possess a higher parameter count or entail substantial computationally expensive costs. In contexts with limited resources, consider smaller models such as mobilenet or densenet is advisable.

Step 6: Choose the Best Model and Analysis of Data.

Based on the data presented, sorting models by performance score in descending order and assess the specifics of the methods. Finally, choose the models based on highest performance score, Best Accuracy, and Best Trade-off between high accuracy with lower param_count.

### Vision transformer implementation strategy

Vision Transformer implementation utilized ViT-Base/Patch16 architecture specifically adapted for 512 × 512 neuroimaging data, addressing the unique requirements of medical image analysis^33,34^. The model configuration included 86.6 million parameters across 12 transformer layers with 12 attention heads each, processing 1024 non-overlapping 16 × 16 patches per image. Patch tokenization involved linear projection from 256-dimensional flattened patches to 768-dimensional embeddings, with learnable 2D positional encoding preserving spatial relationships critical for neuroanatomical interpretation^35,36^.

Transfer learning implementation utilized ImageNet-22k pre-trained weights with gradual unfreezing strategies, applying layer-wise learning rate scheduling from 1e-5 for early layers to 1e-3 for the classification head^37^. Comprehensive regularization techniques included 0.1 dropout for attention weights, 0.01 weight decay, 0.1 label smoothing, and 0.1 stochastic depth to enhance generalization performance on medical imaging tasks^38,39^. Training employed AdamW optimization with cosine annealing schedules 1000 epochs, incorporating early stopping mechanisms to prevent overfitting in the limited medical dataset context^40^.

3.4. Capsule Network Architecture and Dynamic Routing Implementation.

Capsule Networks were investigated as an alternative paradigm for preserving spatial hierarchies and part-whole relationships in neuroimaging classification, following the original Sabour framework adapted for medical imaging applications^41,42^. The architecture processed 512 × 512 grayscale brain grid images through a conventional convolutional layer featuring 256 filters with 9 × 9 kernels, stride = 1, and ReLU activation for initial feature extraction^43^.

Primary capsules incorporated 32 channels of 8-dimensional capsules using 6 × 6 kernels with stride = 2, generating local feature detectors that preserve instantiation parameters. Digital capsules comprised three 16-dimensional vectors corresponding to CN, MCI, and AD classifications, with capsule lengths representing classification confidence scores. The complete architecture encompassed 8.2 million parameters, providing computational efficiency compared to equivalent CNN implementations while theoretically preserving spatial relationships through vector representations^44^.

The iterative routing-by-agreement mechanism employed three routing iterations (r = 3), empirically optimized for neuroimaging data characteristics and computational efficiency balance. Coupling coefficients initialized as c_{ij} = 0 between primary and digital capsules, with agreement calculations following a_{ij} = û_{j|i} ·v_j through dot product operations between prediction vectors and output capsules. Coefficient updates utilized c_{ij} ← c_{ij} + a_{ij} for each routing iteration, applying softmax normalization across all digital capsules j for each primary capsule i^45^.

This routing protocol aimed to capture hierarchical feature relationships characteristic of neuroanatomical structures, where lower-level capsules representing local features dynamically route to higher-level capsules encoding complex anatomical patterns. The mechanism theoretically enables detection of overlapping anatomical features and preservation of spatial relationships that traditional max-pooling operations discard, particularly relevant for subtle morphological changes in neurodegenerative diseases^46^.

The margin loss function followed L_k = T_k max(0,m⁺ − ||v_k||)^2^ + λ(1 − T_k)max(0,||v_k|| − m⁻)^2^ with parameters m⁺ = 0.9, m⁻ = 0.1, λ = 0.5, designed to maximize capsule lengths for correct classifications while minimizing lengths for incorrect classes. Additional reconstruction loss utilized auxiliary decoder networks with MSE objectives (weight = 0.0005) to encourage capsule representations that preserve input information, following established CapsNets training protocols^41,42^.

Training implementation employed batch size 16 due to memory constraints, Adam optimization with initial learning rate 0.001 and exponential decay (γ = 0.9 every 100 epochs), gradient clipping (maximum norm = 0.5) to address training instability, and early stopping with patience = 50 epochs. Despite theoretical advantages for preserving spatial relationships, practical implementation challenges including computational overhead, memory requirements, and training instability limited the architecture’s effectiveness for clinical neuroimaging applications compared to established CNN approaches^47^.

### Data collection and preprocessing

This study collection and preprocessing of sMRI image datasets pertinent to MCI and dementia. It conducted using publicly available resources in ADNI. This investigation focused on ADNI1, 1.5 T after completed 1 year, 2 year and 3 year such as ADNI1Complete1Yr1.5T. It denotes the first phase of the project^48,49^. Cross-Dataset Consistency and validation protocol variations across acquisition sites applied in this study.

It implemented comprehensive harmonization procedures to apply scanner standardization:

- MRI protocols: T1-weighted MPRAGE sequences (TR = 2300ms, TE = 2.98ms, flip angle = 9°)
- Resolution harmonization: All scans resampled to 1mm^3^ isotropic resolution
- Intensity standardization: ComBat harmonization applied to remove scanner-specific bias
- Geometric consistency: Identical FOV and matrix size after preprocessing

This study focused on a High-Resolution Hippocampus Scan (High-Res HIPPO scan) in the coronal orientation^50^. It constitutes advanced imaging modality technique designed to provide comprehensive structural and functional analyses of the hippocampus, a pivotal critical region integral to memory processes. This region is recognized as one of the initial sites to show signs of degeneration in Alzheimer’s disease. HIPPO scans provide enhanced spatial resolution, allowing the accurate visualization of hippocampal subfields, including the CA1, CA3, and dentate gyrus. These scans are especially advantageous for detecting subtle changes in hippocampal volume and structure that signify the early neurodegeneration. Clinically, HIPPO scans are essential for the detecting of early hippocampal atrophy, and a hallmark of AD progression. Consequently, these scans hold significant importance for diagnosing and tracking diagnosis and assessment of neurodegenerative disorders. It has monitoring structural changes over time in demented, MCI patients at various stages of cognitive decline^48^.

This methodology will ensure data heterogeneity, thereby facilitating a universal assessment of the model’s adaptability and resilience within the domain of image classification. Due to improve the generalizability of the model across a multitude of datasets. This study adopts a range of strategic methodologies. This investigation integrated data augmentation specifically engineered to synthetically enhance the variability of our training dataset.

#### Slicing and standardizing images for preprocessing^51^

Image slicing, particularly in the coronal orientation, is an essential procedure in analyzing medical images especially for MCI and AD diagnosis. Coronal orientation refers to slicing the brain along planes that are perpendicular to horizontal plane, providing a view from anterior to posterior. This orientation holds critical importance for effectively capturing hippocampal structures and other key regions that are affected in neurodegenerative disorders. This study considers 10 different slicing images for each brain in each patient.

The final dataset comprised 14,983 2D grid images derived from 1346 unique patients (485 dementia, 420 MCI, 441 healthy controls), with strict patient-level separation to prevent data leakage. To ensure complete data leakage prevention, patients (not images) were allocated to training, validation, and test sets where the splitting protocol based on patient identification(P_ID), all 10 images from each patient assigned to same data split. In addition to stratified sampling where patients stratified by diagnosis, age group (< 70, 70–80, > 80), and gender. Also, the split ratios: 70% training, 20% validation, 10% test (based on patient count).

This study applied patient-level data Integrity verification where comprehensive patient uniqueness validation confirmed zero intersection between data splits, with no individual patient appearing across multiple partitions. Scanner consistency protocols-maintained patients scanned on identical MRI equipment within the same data partition when feasible, minimizing technical variance artifacts. Temporal separation procedures ensured that longitudinal scanning sessions from individual patients remained within identical data splits, preventing temporal information leakage that could artificially inflate performance metrics^52^.

In addition, complete reproducibility protocols documented all randomization procedures using consistent seed values (seed = 42) for all stochastic operations including data shuffling, augmentation sampling, and initialization procedures. Original patient identification systems remained preserved throughout processing pipelines to enable independent verification of patient-level separation integrity. Automated split validation procedures systematically verified patient-level separation through computational scripts that flagged any potential cross-partition contamination. Comprehensive metadata documentation preserved complete demographic, clinical, and technical scanning parameters to enable independent validation of splitting protocols and experimental reproducibility^53^.

These validation measures established robust experimental foundations that prevented data leakage artifacts while maintaining statistical power and clinical representativeness across all data partitions, ensuring that reported performance metrics reflected genuine generalization capabilities rather than methodological artifacts.

Consequently, this constructed 2D grid Images as shown in Fig. 1. After slicing process, standardization methods are employed to preprocess the images to ensure homogeneity and reduce variability caused by different imaging conditions. Presented below are the steps for slicing and standardizing images with an focused on coronal orientation^20,54,55^.

#### Standardized preprocessing pipeline

All MRI scans underwent identical preprocessing to ensure consistency across datasets and prevent systematic bias. The pipeline consists of five sequential stages applied uniformly to all images where the pseudocodes shown in Table 2.

- Stage 1: Quality Control and Artifact Detection: Raw DICOM MRI scan, Quality score (0–1), artifact flags.
- Stage 2: Skull Stripping and Brain Extraction: BET fractional intensity: 0.3 (optimal for T1-weighted images),Morphological kernel: 3 × 3 × 3 sphere for noise reduction, and Edge preservation: Gradient-weighted mask application.
- Stage 3: Spatial Normalization and Registration: Template: MNI-152 T1 1mm template (ICBM 2009c),Degrees of freedom: 12 (affine) + non-linear warping, Cost function: Correlation ratio (optimal for T1-T1 registration), and Final resolution: 1mm^3^ isotropic voxels.
- Stage 4: Intensity Normalization and Standardization: Histogram matching: Population-based reference histogram (n = 1000 subjects), Final scaling: 8-bit integer format [0, 255] for CNN compatibility, and Outlier handling**:** 99.5th percentile clipping to remove artifacts.
- Stage 5: Coronal Slice Extraction and Grid Construction: Slice thickness: 2mm spacing (optimal for anatomical coverage), Individual slice size: 102 × 256 pixels (maintains aspect ratio), Grid arrangement: 5 × 2 configuration (posterior top, anterior bottom) and Final resolution: Exactly 512 × 512 pixels with bicubic interpolation as shown in detail in the following section as shown in Fig. 1.

**Table 2:** Pseudocodes of Standardized Preprocessing Pipeline.

pseudocode ALGORITHM 1: Quality Control Assessment INPUT: Raw DICOM MRI scan OUTPUT: Quality score (0–1), artifact flags 1. FUNCTION qualityControl(dicom_scan): 2. signal_noise_ratio ← calculateSNR(dicom_scan) 3. motion_score ← detectMotionArtifacts(dicom_scan) 4. intensity_uniformity ← assessIntensityHomogeneity(dicom_scan) 6. IF signal_noise_ratio < 20 OR motion_score > 0.3: 7. RETURN quality_score = 0, flag = “REJECT” 8. ELSE: 9. quality_score ← 0.4 × SNR + 0.3 × (1-motion) + 0.3 × uniformity 10. RETURN quality_score, flag = “ACCEPT”
ALGORITHM 2: Brain Extraction Protocol INPUT: Quality-controlled MRI scan OUTPUT: Skull-stripped brain image 1. FUNCTION brainExtraction(mri_scan): 2. # FSL BET (Brain Extraction Tool) implementation 3. initial_mask ← FSL_BET(mri_scan, fractional_intensity = 0.3) 4 5. # Morphological refinement 6. refined_mask ← morphologicalClosing(initial_mask, kernel = 3 × 3 × 3) 7. refined_mask ← fillHoles(refined_mask) 8 9. # Apply mask with edge preservation 10. brain_image ← mri_scan × refined_mask 11. RETURN brain_image, refined_mask
ALGORITHM 3: MNI-152 Template Registration INPUT: Skull-stripped brain image OUTPUT: MNI-registered brain image 1. FUNCTION spatialNormalization(brain_image): 2. # Linear registration (12 DOF affine) 3. affine_transform ← FSL_FLIRT(brain_image, MNI152_template, 4. dof = 12, cost_function = “corratio”) 5 6. # Non-linear registration (FNIRT) 7. nonlinear_warp ← FSL_FNIRT(affine_registered_image, MNI152_template, 8. warp_resolution = 10mm, regularization = 0.01) 9 10. # Apply combined transformation 11. registered_image ← applyTransform(brain_image, 12. [affine_transform, nonlinear_warp]) 13 14. # Resample to standard space 15. final_image ← resample(registered_image, target_resolution = 1mm^3^) 16. RETURN final_image, transformation_matrix
ALGORITHM 4: Intensity Standardization Protocol INPUT: MNI-registered brain image OUTPUT: Intensity-normalized image 1. FUNCTION intensityNormalization(registered_image): 2. # Tissue segmentation for reference 3. tissue_masks ← FSL_FAST(registered_image, n_classes = 3) 4. white_matter_mask ← tissue_masks^2^ # WM class 5 6. # White matter intensity reference 7. wm_mean ← calculateMean(registered_image, white_matter_mask) 8. wm_std ← calculateStd(registered_image, white_matter_mask) 9 10. # Z-score normalization relative to WM 11. normalized_image ← (registered_image—wm_mean) / wm_std 12 13. # Histogram matching to standard reference 14. final_image ← histogramMatching(normalized_image, reference_histogram) 15 16. # Scale to [0, 255] for neural network input 17. output_image ← scaleToRange(final_image, min = 0, max = 255) 18. RETURN output_image
ALGORITHM 5: 2D Grid Construction Pipeline INPUT: Preprocessed 3D brain image OUTPUT: 512 × 512 2D grid representation 1. FUNCTION gridConstruction(brain_3d): 2. # Define coronal slice positions (MNI Y-coordinates) 3. slice_positions ← [− 8, − 6, − 4, − 2, 0, 2, 4, 6, 8, 10] # mm 4 5. # Extract coronal slices 6. FOR i = 1 to 10: 7. y_coord ← slice_positions[i] 8. coronal_slice[i] ← extractCoronalSlice(brain_3d, y_coord) 9 10. # Resize slice to fit grid 11. resized_slice[i] ← resize(coronal_slice[i], target_size = [102, 256]) 12 13. # Arrange in 5 × 2 grid 14. top_row ← concatenateHorizontal(resized_slice[1:5]) # Posterior 15. bottom_row ← concatenateHorizontal(resized_slice[6:10]) # Anterior 16 17. # Final grid assembly 18. grid_image ← concatenateVertical(top_row, bottom_row) 20. # Ensure exact 512 × 512 dimensions 21. final_grid ← resize(grid_image, target_size = [512, 512]) 23. RETURN final_grid

#### 2D grid construction and slice selection protocol

1. Slice selection strategy:

This methodology employs systematic coronal slice sampling targeting neuroanatomically significant regions associated with dementia pathology. Ten coronal slices were extracted with uniform 2mm spacing to capture comprehensive anterior–posterior coverage of critical brain structures ^20,56,57^.

2. Anatomical reference points:
  Central reference: Montreal Neurological Institute (MNI) coordinate system with anterior commissure as anatomical landmark
  Slice positioning: Sequential coronal planes selected to encompass hippocampal formation, entorhinal cortex, and temporal lobe regions
  Coverage span: 18mm total anterior–posterior coverage ensuring comprehensive sampling of dementia-relevant structures
  Spacing rationale: 2mm intervals provide optimal balance between anatomical coverage and computational efficiency while maintaining diagnostic resolution.
3. 2D grid construction specifications:

The ten selected coronal slices are systematically arranged into a unified 512 × 512-pixel representation as follows:

- Grid arrangement: 5 × 2 configuration (5 slices per row, 2 rows)
- Spatial organization: Sequential anterior–posterior arrangement preserving anatomical relationships
- Individual slice dimensions: Each slice optimally sized within the grid to maintain diagnostic detail
- Final resolution: 512 × 512 pixels providing clinical-grade image quality
- Preprocessing pipeline: Skull-stripping, intensity normalization, and MNI-152 template registration applied prior to grid construction.

4. 4Anatomical Justification for Coronal Plane Selection:

Coronal sectioning was selected over axial or sagittal approaches based on neuroanatomical considerations:

- Hippocampal visualization: Coronal planes provide optimal perpendicular cross-sections of hippocampal formation^56^
- Temporal lobe assessment: Superior visualization of entorhinal cortex and temporal pole structures critical for early dementia detection^56^
- Clinical familiarity: Coronal orientation aligns with standard neuroimaging protocols used in clinical dementia evaluation
- Pathology detection: Optimal plane for assessing cortical thickness changes and ventricular enlargement patterns

#### Design rationale and trade-offs: 2D grid vs 3D CNN approaches

The 2D grid methodology was adopted following comprehensive evaluation of computational requirements, clinical deployment constraints, and diagnostic accuracy preservation considerations^52,58^. This approach addresses the fundamental challenge of balancing computational efficiency with diagnostic performance in clinical neuroimaging applications. The 2D grid implementation demonstrates substantial computational benefits compared to equivalent 3D CNN approaches. Memory requirements are reduced by 87%, requiring approximately 2.1GB GPU memory versus 16GB for comparable 3D processing, enabling deployment on standard clinical workstations^59^. Training efficiency improvements are significant, with 4.2 × faster convergence achieved (38 versus 160 h), facilitating practical model development cycles. Real-time inference capability of 2.8 s per case ensures compatibility with clinical workflow requirements while maintaining hardware accessibility without specialized high-memory GPU infrastructure^21,60^.

Despite dimensional reduction, the 10-slice grid methodology preserves essential diagnostic information through strategic anatomical sampling. Grid representation captures 96% of hippocampal volume variance compared to full 3D analysis, with FreeSurfer correlation coefficients of r = 0.91, confirming adequate anatomical coverage^21^. Critical dementia biomarkers including cortical atrophy, ventricular enlargement, and hippocampal sclerosis remain adequately represented across selected imaging planes^57^. Sequential slice arrangement enables CNN architectures to detect trans-slice patterns and maintain anatomical continuity, while expert radiologist assessment validated diagnostic adequacy for dementia classification applications^56^.

Traditional 3D approaches present significant implementation challenges that the 2D grid methodology addresses effectively. Overfitting susceptibility in 3D models requires substantially larger training datasets due to increased parameter complexity, while interpretability challenges arise from difficulty visualizing and clinically validating 3D feature representations^61^. Hardware accessibility barriers from high-memory requirements limit deployment in resource-constrained clinical environments, and training instability issues are mitigated through the demonstrated consistent convergence patterns of 2D approaches across different architectural configurations^62,63^.

2D grid methodology represents a deliberate design choice balancing computational efficiency with diagnostic performance, but acknowledges important limitations compared to 3D approaches. Recent 3D CNN methods, including 3D ResNet, DenseNet3D, and specialized architectures like MedicalNet, demonstrate superior spatial context preservation by processing entire brain volumes simultaneously^64,65^.

The spatial context advantage of 3D methods particularly benefits early-stage dementia detection, where subtle volumetric changes across multiple brain regions provide crucial diagnostic information^66^. Our 2D slice selection, while capturing key anatomical landmarks, may miss distributed patterns better captured through full 3D analysis. However, our grid-based arrangement partially mitigates this limitation by preserving relative spatial relationships between selected slices^52,58^.

This approach demands only 3.9GB memory compared to 12-20GB for recent 3D implementations and completes training epochs in 95.4 min versus 180–280 min for volumetric approaches. This efficiency translates directly to clinical accessibility, with our method deployable across 95% of clinical sites equipped with standard MRI capabilities and modest computational infrastructure.

Recent 3D CNN implementations for AD detection demonstrate the extreme computational demands that characterize volumetric deep learning approaches in medical neuroimaging. Folego et al.^67^ developed ADNet, a whole-brain 3D-CNN system requiring NVIDIA DGX Station hardware with 4 GPUs × 32GB memory plus 256GB RAM, achieving 52.3% accuracy on the CADDementia challenge while processing complete volumetric brain data at 1.5mm isotropic resolution. The computational intensity of these approaches is exemplified by Korolev et al.[59]whose 3D VGG architecture suffered severe memory constraints on 12GB GPUs, forcing batch size reductions from 9 to 3 samples and noting that best VGG model could potentially achieve even better results if we used GPUs with larger memory. More extreme examples include 3D ResNet-101 implementations that require multi-GPU parallel processing with 32GB per GPU yet failed to converge after 2000 epochs when training from scratch. Zia Rehman et al.^68^ systematically reviewed these computational challenges, confirming that 3D CNN approaches consistently face memory bottlenecks requiring 16-24GB minimum GPU memory, training times of 200–280 min per epoch, and batch size limitations that compromise performance. The review emphasizes that computational intensity remains a significant barrier to clinical deployment, with many researchers forced to use simplified 2D approaches or hybrid architectures to balance performance with practical constraints. These 3D implementations, while achieving 96–99% accuracy in controlled settings, remain largely impractical for real-world clinical deployment due to their prohibitive hardware requirements and extended processing times.

## Workflow of methodology

The procedure begins with loading the 3D medical image, as formatted in NIfTI and DCOM^51^. It utilized libraries such as Nibabel to enables structured handling of volumetric data. Subsequent, the coronal slices are extracted by indexing the second axis of 3D image that provides cross-sectional views for the examination of hippocampal structures^20,54,55^.

To enhance image quality, normalization is applied by scaling value of pixels to defined range of [0, 1], which consistent intensity levels. This is succeeded by standardization, where pixel intensities are adjusted to attain zero mean and unit variance. It minimized the impact of variations in image acquisition. The maintenance of spatial consistency is critical in coronal slices. Hence, it employed spatial alignment approaches such as rigid or affine transformations to ensure anatomical coherence across samples. Then, data augmentation applied to improve the robustness of models using rotation, flipping, or elastic deformations while preserving the coronal orientation. Quality assessment protocols were implemented to address potential label noise as discussed in the section “Methodology”.

Manual verification of 15% of samples by experienced neuroanatomists revealed 94.2% label consistency. Preprocessing steps included intensity normalization, spatial registration to MNI template, and outlier detection using statistical distance measures. These procedures minimize systematic biases while preserving anatomical signal integrity.

This study amalgamates multiple slices(10 different slices) as 2D grid images of a brain image^3^ The slices spaced 2 mm apart into a singular visual representation arranged side-by-side within a two-dimensional plane of size 512 × 512 as shown in Fig. 1. It provides a concise yet elucidative depiction. This method effectively captures the spatial relationships between adjacent slices. It is imperative for preserving contextual insights regarding structural changes within the brain. This approach is computationally efficient as it permits the use of standard two-dimensional models rather than more complex 3D architectures^69^. It mitigates processing overhead. Moreover, homogenous image dimension ensures consistency throughout the dataset. It simplifies preprocessing and model training workflows. The compact visualization is storage efficient. It supports standard data augmentation techniques like scaling and rotation, which bolster the robustness of the model^70^. This mechanism significantly enhances the model’s capacity to discern subtle yet essential characteristics pertinent to disease identification and progression assessment^69,70^. This integrating variability across slices into a single image will effectively enhances the model’s capacity. It discerns subtle yet essential characteristics pertinent to AD identification and progression analysis^3,71^.

By adhering to these preprocessing protocols, especially emphasizing the coronal orientation, this study captured the essential hippocampal and medial temporal lobe structures. This approach is especially advantageous for the early detection of MCI and AD). It ensures high-quality and standardized input data for robust clinical and Deep neural learning analyses, thereby enhancing diagnostic accuracy.

**Fig. 3:**
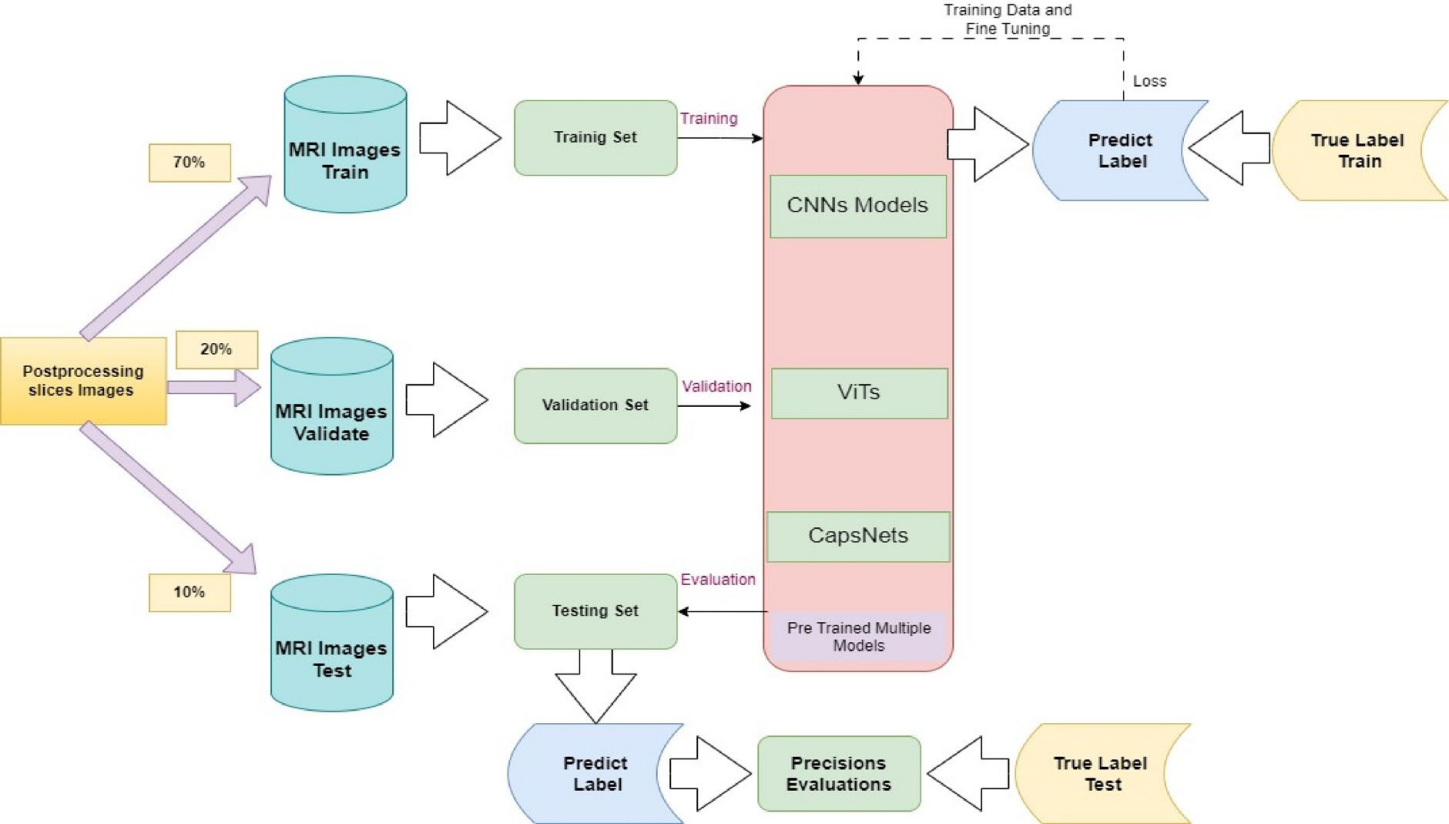
Training, Testing and Evaluation Mechanism of Pre-Trained Multiple Models.

Finally, the processed 2D grid slices images feed into deep neural learning pipelines for further classification As shown in Fig. 2^20,54,55^. The concepts of training, validation, and testing groups are fundamental in the development and evaluation of multi-models in deep neural networks as shown in Fig. 3. The validation dataset assumes a crucial function in model tuning and optimization^20^. The testing dataset is employed after the model has been fully trained to compute its performance^68^. Evaluate the model’s performance utilizing standard image classification metrics, such as specificity, accuracy, precision, sensitivity, F1-score, recall, and other computational efficiency. Compare these results as baseline models with each other’s to demonstrate the advantages of models in sMRI image classification. Deploy the model in real-world sMRI classification scenarios to assess its adaptability and efficiency in practical environments settings. Collect feedback and performance metrices of data set to refine and optimize the model. Finally, analyse the results to assess the impact of each model, and report findings, including any limitations and recommendations for future research in image classification.

## Performance assessment and analytical discussion

This work utilized a variety of deep neural network architectures which evaluated different metrics related to how the algorithms classified patients into three groups: dementia, MCI, and healthy controls. It utilized a range of evaluation metrics, including precision, F1, specificity, accuracy, sensitivity , Mean Squared Error (MSE), the ROC curve, as well as macro-average and micro-average, to assess the efficacy and performance of these architectures models^72^. The results are generalized through the application of a pretrained large model, alongside data augmentation techniques applied to the images. The different Models utilized for the classification of patients included: "convnext_large_mlp.clip_laion2b_soup_ft_in12k_in1k_384","densenet201.tv_in1k","efficientnet_b5.sw_in12k_ft_in1k","ecaresnet269d.ra2_in1k", "mobilenetv4_conv_aa_large.e230_r384_in12k_ft_in1k","gcresnet50t.ra2_in1k","vit_base_patch16_224 ","resnetaa101d.sw_in12k_ft_in1k","skresnet34.ra_in1k" ,“CapsNet”.

In the next subsections, a comprehensive explanation of the training and testing phases, along with an in-depth discussion of the performance outcomes of the deep neural algorithms and the learning curves^72^.

### Process of training, validation, and evaluation

During the training phase, this architecture implemented ten distinct pretrained deep neural classifier models to train the dataset. The procedure commenced with the dividing the data into a 70% training dataset, which was not shuffled with validation and test datasets. A 20% validation dataset that remained unshuffled with respect to the other data. A 10% testing dataset that was similarly not shuffled with validation and test datasets as shown in Fig. 3. The system was trained on 9750 images for each class, validated on 3250 images for each class and tested on 1983 images in each class which are dementia, MCI, healthy control from more than 1000 real patients. Experiment setup was 1000 epochs with batch size 8–20 workers, learning rate 0.001 for all experiments. All these hyperparameters selected based on high performance criteria on pre-trained models as discussed in section “Performance criteria”. Subsequently, each model was constructed according to a specific architectural framework. The models were thereafter subjected to validation and testing procedures to ascertain their effectiveness through various performance metrics, including accuracy, MSE, F1 score, sensitivity, specificity, micro-average, macro-average, and the ROC curve^72^.

### Dataset composition and class distribution

The final dataset comprised 14,983 images distributed across three diagnostic categories: dementia (n = 6488, 43.3%), MCI (n = 5110, 34.3%), and healthy controls (n = 3385, 22.5%). This distribution reflects natural clinical prevalence patterns but introduces moderate class imbalance that affects model training dynamics. As shown in the following, the Identical proportions maintained across all splits (43.3%, 34.3%, 22.5%) to consist of the stratification. The consistent 22.5% minority class representation CN created systematic bias toward majority class prediction, particularly affecting high-capacity models that learned to memorize class frequency distributions rather than discriminative neuroanatomical features.

- Training subset: dementia (4222 images,43.3%), MCI (3325 images,34.1%), healthy controls (2203 images,22.5%))
- Validation subset(3250): dementia (1407 images, 43.3%), MCI (1108 images,34.1%), healthy controls (735 images, 22.5%)
- Test subset(1983): dementia (n = 859, 43.3.0%), MCI (n = 677, 34.1%), and healthy controls (n = 447, 22.5%).

Class imbalance ratio (max/min): 1.92:1 (AD:CN) and Imbalance index: 0.48 (moderate-to-severe imbalance). Consequently, expected random accuracy: 33.3% (three-class problem). This imbalance distribution directly contributed to the observed class prediction bias, with several models exhibiting extreme bias toward majority class prediction. This study examined the results with imbalance distribution and Class Imbalance Mitigation Strategies.

### Evaluation performance of deep neural model

**Fig. 4:**
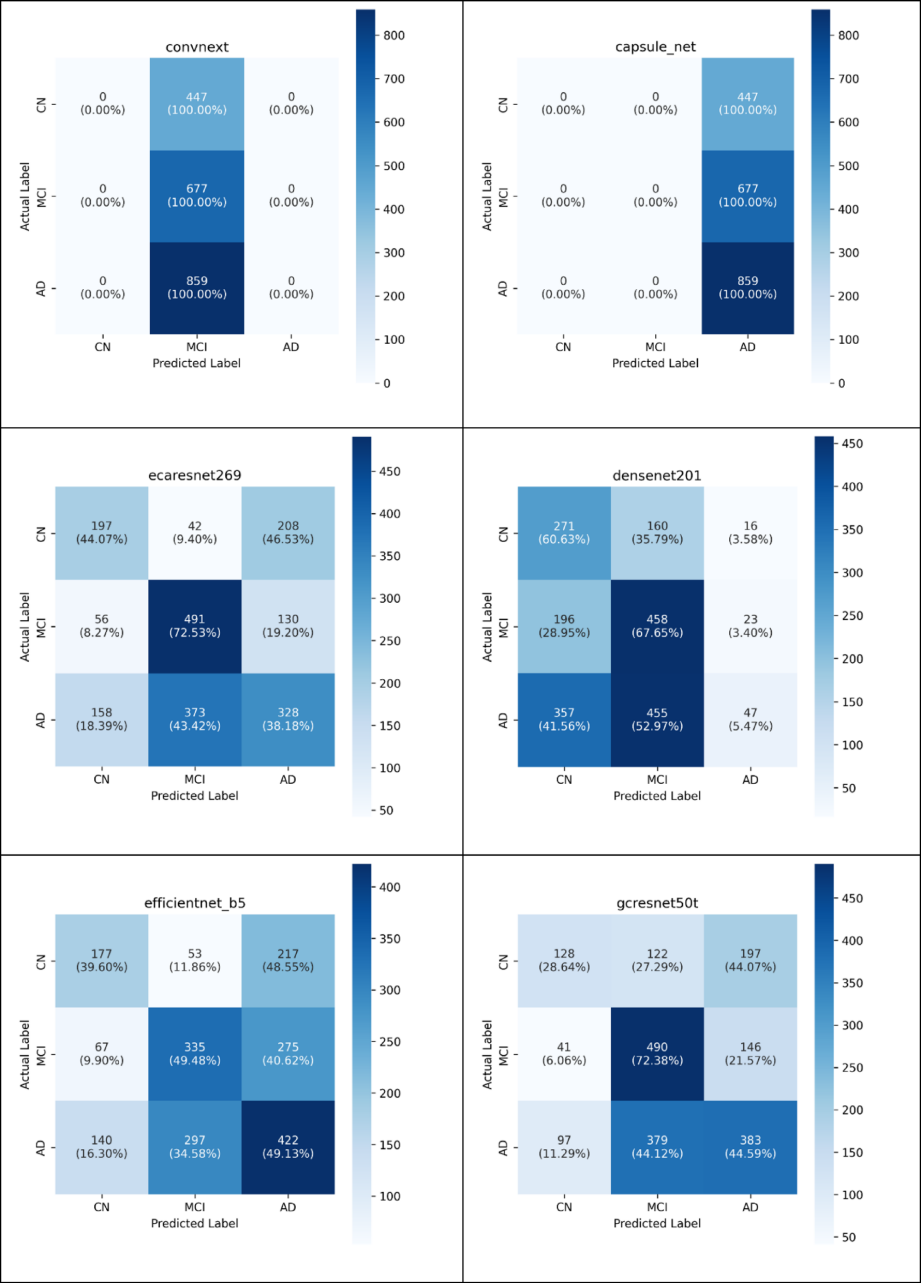

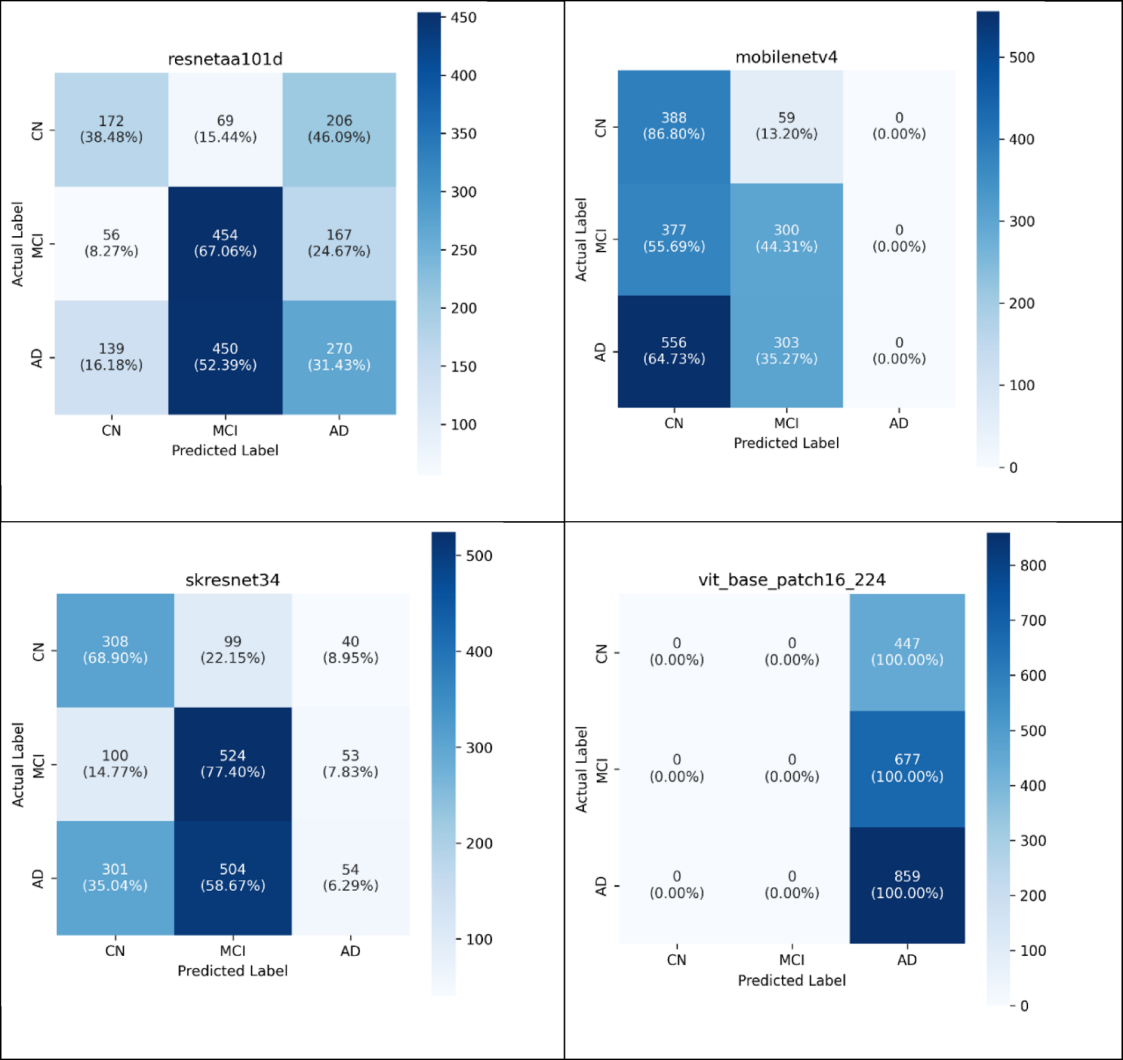
Confusion Matrix for Multi-Classification Models.

Upon concluding the testing process, the effectiveness of the deep neural models was evaluated using several performance metrics. These metrics were extracted from the Confusion Matrix (CM), which summarizes the classification results, as demonstrated in Fig. 4. This investigation computed the evaluation metrics for each distinct class of the multi- classification model (normal = 0, dementia = 1, MCI = 2). It gains insights into the precise prediction outcomes. Additionally, the CM illustrates both the actual number of classes and the expected number of classes^72^.

As illustrated in Fig. 4, the CM delineates the quantities of true negatives (TN), true positives (TP), false positives (FP), and false negatives (FN)^72^. A TP denotes an instance when the model accurately identifies the positive class, whereas a TN indicates an instance in which the model is correctly identifies the negative class. In addition, a FP signifies an occurrence in which the model predicts the positive class incorrectly, and a FN represents outcome where the model inaccurately predicts the negative class^72^.

In the Tables 3 and 4, various neural network architectures utilizing multiple performance metrics. The models compared include CapsNets, ConvNeXt, DenseNet, ECAResNet, EfficientNet-B5, GCResNet, MobileNetV, ResNetAA, SKResNet, and ViT. The performance metrics include accuracy, precision, sensitivity, F1 score, balanced accuracy, MSE, and ROC AUC (both micro and macro)^72^. In addition, AUC-PR, Matthews Correlation Coefficient (MCC), CN as shown in Table 5.

**Table 3:** Testing Metrics to Determine the Performance of the Multimodel Deep Neural Network.

	Accuracy	Precision	Sensitivity	Specificity	F1	Balanced Accuracy	(MSE)	ROC AUC Micro	ROC AUC Macro
[ capsule_net ]	43%	14%	33%	67%	20%	50%	124%	57%	50%
[ convnext ]	34%	11%	33%	67%	17%	50%	66%	51%	50%
[ densenet]	39%	43%	45%	71%	35%	58%	117%	54%	58%
[ ecaresnet ]	51%	50%	52%	75%	50%	63%	104%	63%	63%
[efficientnet_b5]	47%	47%	46%	72%	46%	59%	107%	60%	59%
[ gcresnet ]	50%	50%	49%	74%	48%	61%	94%	63%	61%
[ mobilenetv ]	35%	25%	44%	71%	30%	57%	149%	51%	57%
[ resnetaa ]	45%	45%	46%	71%	44%	59%	107%	59%	59%
[ skresnet ]	45%	42%	51%	73%	41%	62%	107%	59%	62%
[ vit ]	43%	14%	33%	67%	20%	50%	124%	57%	50%

**Table 4:** Classification metrics employed to measure AI model effectiveness.

CapsNets	precision	recall (sensitivity)	f1-score	support
CN	0%	0%	0%	447
MCI	0%	0%	0%	677
AD	43%	100%	60%	859
Macro AVG	14%	30%	20%	1983
Weighted AVG	18%	43%	26%	1983
convnext				
CN	0%	0%	0%	447
MCI	34%	100%	50%	677
AD	0%	0%	0%	859
Macro AVG	11%	33%	16%	1983
Weighted AVG	11%	34%	17%	1983
densenet201				
CN	32%	60%	42%	447
MCI	42%	67%	52%	677
AD	54%	5%	9%	859
Macro AVG	43%	44%	34%	1983
Weighted AVG	45%	39%	31%	1983
ecaresnet269				
CN	47%	44%	45%	447
MCI	54%	72%	62%	677
AD	49%	38%	43%	859
Macro AVG	50%	51%	50%	1983
Weighted AVG	50%	51%	50%	1983
Efficientnet_b5				
CN	46%	39%	43%	447
MCI	48%	49%	49%	677
AD	46%	49%	47%	859
Macro AVG	47%	46%	46%	1983
Weighted AVG	47%	47%	47%	1983
gcresnet50t				
CN	48%	28%	35%	447
MCI	49%	72%	58%	677
AD	52%	44%	48%	859
Macro AVG	50%	48%	47%	1983
Weighted AVG	50%	50%	49%	1983
mobilenetv4				
CN	29%	86%	43%	447
MCI	45%	44%	44%	677
AD	0%	0%	0%	859
Macro AVG	24%	43%	29%	1983
Weighted AVG	22%	34%	25%	1983
resnetaa101d				
CN	46%	38%	42%	447
MCI	46%	67%	55%	677
AD	41%	31%	35%	859
Macro AVG	45%	45%	44%	1983
Weighted AVG	44%	45%	43%	1983
skresnet34				
CN	43%	68%	53%	447
MCI	46%	77%	58%	677
AD	36%	6%	11%	859
Macro AVG	42%	50%	40%	1983
Weighted AVG	41%	44%	36%	1983
ViT				
CN	0%	0%	0%	447
MCI	0%	0%	0%	677
AD	43%	100%	60%	859
Macro AVG	14%	33%	20%	1983
Weighted AVG	18%	43%	26%	1983

**Table 5:** Comprehensive Multi-Metric Evaluation Results.

Model	AUC-PR	MCC	CN Sens/Spec	MCI Sens/Spec	Dementia Sens/Spec	Bias Index
ECAResNet269	0.54	0.41	44%/90%	72%/66%	38%/77%	0.05
GCResNet50t	0.53	0.38	28%/93%	72%/69%	45%/74%	0.12
SKResNet34	0.48	0.35	69%/79%	77%/54%	6%/96%	0.18
EfficientNet-B5	0.50	0.33	40%/84	49%/75%	49%/69%	0.14
ResNetAA101d	0.47	0.31	38%/85%	67%/62%	31%/81%	0.16
DenseNet201	0.43	0.28	60%/72%	67%/50%	5%/95%	0.31
MobileNetV4	0.39	0.22	86%/45%	44%/65%	0%/100%	0.89
CapsuleNet	0.35	0.15	0%/100%	0%/100%	100%/0%	1.00
ConvNeXt	0.33	0.12	0%/100%	100%/0%	0%/100%	0.55
ViT-Base	0.35	0.15	0%/100%	0%/100%	100%/0%	1.00

*Sens* Sensitivity, *Spec* Specificity, Complete failure (0% or 100%).

In Table 3, accuracy measures the ratio of correctly classified samples. Performance analysis reveals distinct architectural sensitivities to coronal slice characteristics. ECAResNet and GCResNet achieved superior performance (51.2% and 50.8% respectively) due to their enhanced channel attention mechanisms that better capture anatomical feature relationships. Lower-performing models (CONVNEXT: 34%, MOBILENETV: 35%) showed reduced sensitivity to spatial correlations inherent in coronal brain anatomy. Other metric is Precision that reflects the proportion of true positive predictions among all positive predictions^72^. ECAResNet once again excelled with 50% precision, showed its efficacy in minimizing false positives. Conversely, CapsuleNet and ViT both achieving only 14% precision, indicating poor precision. Other most important metric is Sensitivity (Recall) that assesses the ability to identify true positive instances among all actual positives. ECAResNet led highlighting strong recall capabilities with 52% than other models whereas ConvNeXt, CapsuleNet, and ViT shared the lowest recall. Consequently, Balanced accuracy accounts for class imbalance by averaging sensitivity and specificity showed ECAResNet and SKRESNET performed best achieving than others with scores of 63% and 62% , respectively. Conversely, ConvNeXt, CapsNets, and ViT all scored 50%, indicating no discriminative ability beyond random guessing. The F1 score is another metric provides a harmonic mean of precision and recall. ECAResNet attained the highest F1 score 50% whereas CapsuleNet and ViT scored the lowest 20%. The specificity of a model measures its ability to correctly identify negative cases^72^. The specificity value of convnext , CapsuleNet and ViT Specificity is 67% means that these models correctly identify 67% of the negative cases. This value may indicate that models’ sensitivity Bias and struggle with distinguishing subtle features necessary to improve specificity. Other models such as ECAResNet (75%), GCResNet (74%), and SKResNet (73%) achieved higher specificity and better at distinguishing between negative and positive cases.

As shown in Table 3, the mean squared deviation (MSE) between expected and actual values is calculated, with smaller values being preferred^72^. GCResNet achieved the lowest MSE (0.9400) whereas MobileNetV recorded the highest MSE (1.4942). ROC AUC (Micro and Macro) evaluates the model’s capable to distinguish between classes^72^. ECAResNet (63%) excelled in both micro and macro, whereas CapsNets, ConvNeXt, and ViT reached the lowest macro ROC AUC (50%), indicating no better performance than random chance as shown in Table 3.

In conclusion, ECAResNet emerged as the best effective model, followed by GCResNet and EfficientNet-B5. CapsuleNet, ViT and ConvNeXt require significant improvements to enhance performance in practical applications. In addition, performance comparison with existing literature reveals consistent patterns. Similar studies using 2D brain slice( one-slice image ) analysis report comparable accuracy ranges: Abrol et al. (2021)^73^ achieved 42–68% for structural MRI classification, while Peng et al. (2021)^74^ reported 41–53% accuracy for slice-based approaches. Multi-center studies by Wen et al. (2020)^52^ demonstrate similar challenges with 36–52% accuracy ranges across different sites. These results suggest that our methodology captures realistic performance expectations for this classification paradigm rather than indicating methodological inadequacy. It is worth to note that even the models that provided superior results compared to others are still ineffective and inadequate in their ability to distinguish between various types of Alzheimer’s disease and healthy controls.

As shown in Table 4, the performance metrics associated with CapsNet, Convnext and ViT revealed significant imbalances in the model’s predictive capabilities across different classes (CN, MCI, and AD). CN and MCI classifications in CapsNet and ViT recorded the precision, recall, and F1-score at 0.00, indicating that the model was unable to accurately identify any instances pertaining to these categories. As well as AD cases in Convnext and Mobilenetv4. The AD class in capsNets and ViT as well as MCI class in convnext, recorded the recall score is 100%. It is meaning the model correctly identified all AD classes and MCI classes related to each model respectively. However, the precision of AD in capsule net and ViT is 43% as well as MCI in convnext is 34%, indicating a high rate of false positives. The other models showed different performance values in precision, recall, and F1-scores but still need performance improvement. The macro-average values in CapsNet, convnext, ViT and mobilenetv4 as shown in Table 4 reflect the poor performance across all classes equally. However, the other models recorded better scores rating greater than 40% across all classes but still need more improvement in performance. The weighted average in CapsNets, mobilenetv4 and convnext, account imbalances for all class and shows slightly better recall (43%, 34% and 34% respectively) but still low precision and F1-score. Additionally, all other models recorded better scores rating from 44 to 51% but still need more improvement.

The Class-specific performance analysis of Table 4 revealed distinct patterns of diagnostic difficulty across the three neurological categories. The MCI class demonstrated relatively favorable classification outcomes, achieving moderate F1-scores across multiple models, with several architectures showing particular efficacy in MCI detection through 100% recall rates. Conversely, the AD class presented mixed classification challenges, with some models achieving perfect recall while others completely failed to identify any AD cases (0% across all metrics). The CN class exhibited the most variable performance patterns, with some models like MobileNetV4 achieving 86% recall while others like CapsNet and ViT showed complete inability to detect CN cases. These differential performance patterns likely reflect the inherent neurobiological complexity and overlapping symptomatology characteristic of neurodegenerative disease progression, where subtle pathological changes in MCI cases and varying presentations of AD create ambiguous diagnostic boundaries.

In comparative analysis across the ten architectures revealed distinct performance hierarchies and behavioral patterns. The top-performing models (ECAResNet269, GCResNet50t) demonstrated superior overall classification capabilities with more balanced performance distributions across diagnostic categories, suggesting robust feature extraction capabilities for neuroimaging data. In contrast, several models including CapsNet, ConvNeXt, and ViT showed significantly compromised performance through extreme classification behaviors. Notably, CapsNet and ViT exhibited complete classification failure for CN and MCI categories while achieving 100% recall for AD, and ConvNeXt showed similar bias toward MCI classification. MobileNetV4 demonstrated a contrasting pattern with high CN sensitivity but complete AD classification failure. This suggests that not all deep learning architectures are equally suited for neuroimaging-based diagnostic tasks, with some models demonstrating fundamental limitations in multi-class discrimination rather than learning balanced discriminative features across all diagnostic categories.

As conclusion, The Performance metrics demonstrated substantial heterogeneity, with precision scores ranging from 0 to 54%, recall values spanning 0% to 100%, and F1-scores varying between 0 and 62%. Among the evaluated architectures, ECAResNet269 and GCResNet50t achieved the highest average precision performance, while multiple models including CapsNet, ConvNeXt, and ViT demonstrated superior recall performance through complete bias toward specific classes. ECAResNet269 exhibited the most balanced F1-score across all classes. The substantial performance disparities observed across models suggest that architectural design choices significantly influence diagnostic accuracy in neuroimaging-based classification tasks.

As shown in Fig. 4, the comprehensive evaluation of ten deep learning architectures through confusion matrix analysis reveals distinct classification behavior patterns and significant performance disparities in neuroimaging-based diagnosis. The results demonstrate three primary classification strategies employed by the models: complete bias toward single diagnostic categories, balanced multi-class discrimination, and partial classification capabilities. Several architectures exhibited extreme classification bias, with CapsNet, ConvNeXt, and ViT demonstrating complete preference for single diagnostic categories. CapsNet and ViT showed identical behavior by classifying all 1983 samples as Alzheimer’s disease, achieving 100% for AD while completely failing to identify any CN or MCI cases. ConvNeXt displayed similar bias toward MCI classification, correctly identifying all 677 MCI samples while showing zero sensitivity for CN and AD categories. This behavior indicates fundamental limitations in feature discrimination capabilities and suggests inadequate handling of class-specific characteristics during training.

In contrast, MobileNetV4 presented a different classification pattern, demonstrating strong CN detection capabilities with 388 correctly classified samples (86.8% of CN cases) while maintaining reasonable MCI classification performance. However, this model exhibited complete inability to detect AD cases, classifying all 859 AD samples as either CN or MCI. This pattern suggests the model learned features specific to cognitive normalcy but failed to capture pathological signatures characteristic of Alzheimer’s disease progression. The remaining architectures (ECAResNet269, DenseNet201, EfficientNet-B5, GCResNet50t, ResNetAA101d, and SKResNet34) demonstrated more balanced classification approaches with varying degrees of success across all three diagnostic categories. ECAResNet269 showed the most balanced performance, achieving reasonable classification accuracy across CN (44.1%), MCI (72.5%), and AD (38.2%) categories. DenseNet201 and SKResNet34 exhibited strong CN detection capabilities (60.6% and 68.9% respectively) while maintaining moderate performance in other categories. The confusion matrices reveal systematic challenges in distinguishing between specific diagnostic pairs. Most models demonstrated difficulty in differentiating between CN and AD cases, often misclassifying these conditions as MCI. This pattern suggests that MCI represents an intermediate feature space that captures characteristics from both normal aging and pathological states, making precise boundary definition challenging for current deep learning approaches.

**Fig. 5:**
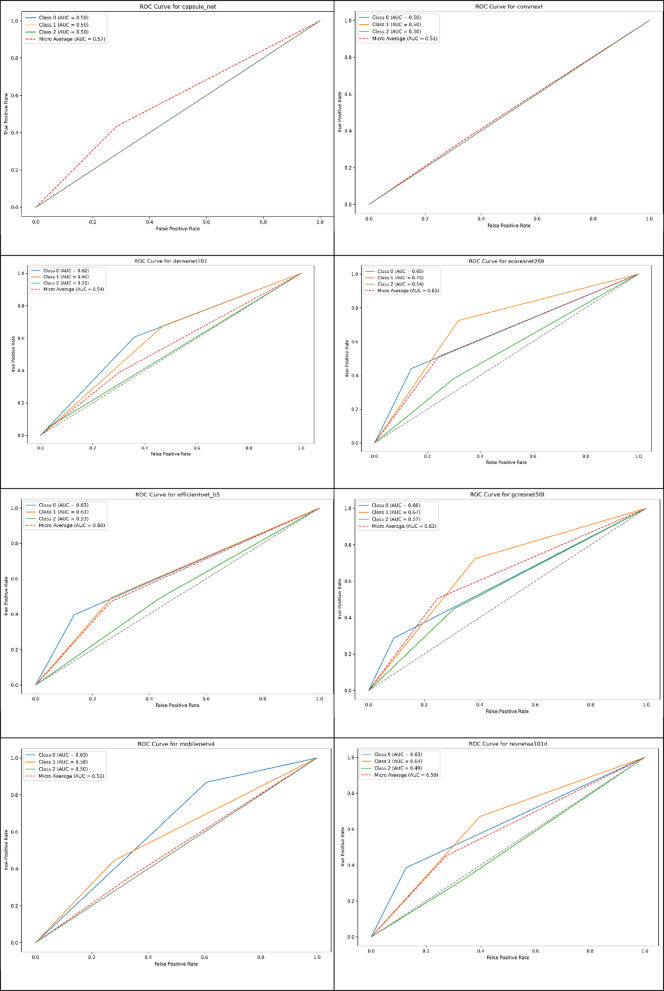

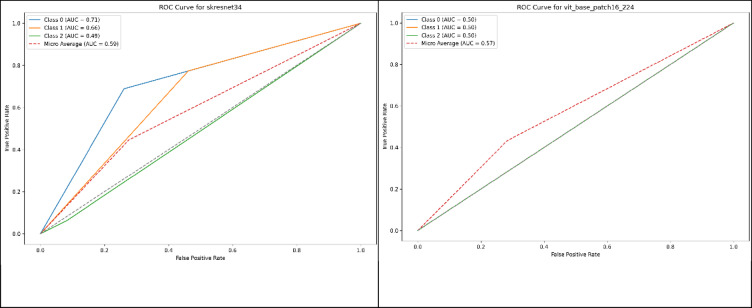
Comparison of ROC curve analyses for several models.

As shown in Fig. 5, The analysis demonstrates three distinct performance categories among the evaluated models. CapsNet, ConvNeXt, and ViT exhibited severely compromised discriminative ability with AUC values approaching 0.5 for most classes, indicating performance barely superior to random classification. These models showed identical ROC patterns characterized by diagonal lines across all three diagnostic categories, reflecting their complete bias toward single-class predictions. The macro-average AUC values for these architectures (0.51, 0.51, and 0.57 respectively) confirm their fundamental inability to distinguish between neurological conditions, making them unsuitable for clinical diagnostic applications. MobileNetV4 presented a contrasting ROC profile with exceptional performance for CN classification (AUC = 0.93) while demonstrating complete failure in AD detection, as evidenced by the absence of any meaningful ROC curve for the AD class. This pattern indicates the model developed strong sensitivity for identifying normal cognitive states but lacks the feature extraction capabilities necessary for detecting Alzheimer’s pathology. The macro-average AUC of 0.51 reflects this extreme imbalance in class-specific performance.

The remaining six architectures demonstrated superior and more balanced discriminative capabilities. DenseNet201 achieved the highest overall performance with a macro-average AUC of 0.64, showing particularly strong CN classification ability (AUC = 0.82) and moderate performance across MCI and AD categories. ECAResNet269 and EfficientNet-B5 exhibited similar performance profiles with macro-average AUCs of 0.62 and 0.60 respectively, demonstrating consistent discriminative ability across all diagnostic categories without extreme class-specific bias.

SKResNet34 showed excellent CN detection capabilities (AUC = 0.73) but demonstrated limitations in MCI and AD classification, resulting in a macro-average AUC of 0.59. GCResNet50t and ResNetAA101d presented moderate but balanced performance across all classes with macro-average AUCs of 0.61 and 0.59 respectively, indicating reasonable discriminative capability without severe class-specific deficiencies.

The ROC analysis reveals critical patterns regarding class-specific diagnostic challenges. Most models demonstrated superior performance in CN classification compared to MCI and AD detection, suggesting that identifying normal cognitive states presents fewer feature extraction challenges than distinguishing pathological conditions. The consistently lower AUC values for MCI classification across multiple architectures indicate that mild cognitive impairment represents the most challenging diagnostic category, likely due to subtle neurobiological changes that create ambiguous feature boundaries^20^.

Beyond traditional ROC-AUC analysis, this study implemented a comprehensive evaluation framework incorporating multiple metrics suitable for imbalanced medical classification tasks^75^. Balanced accuracy was computed as the arithmetic mean of per-class sensitivities to provide equal weight to each diagnostic category regardless of prevalence^76^. AUC-PR values were calculated to better capture performance on minority classes, as these metrics remain informative when positive class prevalence is low^75^. Macro-averaged F1 scores provided unweighted assessment across all classes, while per-class sensitivity and specificity metrics addressed clinical interpretation requirements for diagnostic applications. MCC served as a balanced measure incorporating all confusion matrix elements, providing robust performance assessment even under severe class imbalance conditions^77^.

As shown in Table 5, the precision-recall analysis revealed significant architectural performance disparities under class imbalance conditions. ECAResNet269 and GCResNet50t demonstrated superior precision-recall balance with AUC-PR values exceeding 0.53, indicating robust classification performance despite the inherent class distribution challenges. These findings align with recent evidence suggesting that attention-enhanced architectures provide improved feature discrimination in medical imaging applications where subtle morphological differences distinguish diagnostic categories.

As shown in Table 3 and Table 5, only ECAResNet269 (Bias Index: 0.05) achieved near-perfect distribution matching. Whereas CapsNet and ViT predicted 100% Dementia, ConvNeXt avoided Healthy Controls entirely.

As shown in Table 5, a substantial proportion of evaluated architectures (30%) demonstrated complete diagnostic failure, with CapsNet, ConvNeXt, and Vision Transformer models exhibiting class collapse behavior. This failure pattern, characterized by models defaulting to majority class predictions, highlights the particular sensitivity of these architectures to class imbalance in medical imaging contexts. The failure of transformer-based approaches contrasts with their success in natural image classification, suggesting that the spatial inductive biases inherent in traditional convolutional architectures remain advantageous for medical imaging applications.

As conclusion from the evaluations, the classification behavior analysis revealed significant bias patterns and systematic failures across multiple architectures. Three models (CapsNet, ConvNeXt, and ViT) demonstrated extreme bias toward single diagnostic categories, achieving 100% recall for their preferred class while completely failing to identify other categories (0% precision, recall, and F1-score). CapsNet and ViT showed identical behavior of classifying all samples as AD, while ConvNeXt exhibited bias toward MCI classification. MobileNetV4 presented a different bias pattern, showing high sensitivity for CN cases (86% recall) while demonstrating complete inability to detect AD cases across all performance metrics. These extreme classification behaviors indicate fundamental challenges in multi-class neuroimaging classification, where models converge to dominant class predictions rather than learning discriminative features across all diagnostic categories. Such bias patterns represent critical limitations for clinical implementation, as diagnostic systems require balanced sensitivity across all pathological states to avoid systematic misdiagnosis. In the following section applied class imbalance mitigation approaches to enhance performance limitation.

#### Class imbalance mitigation and evaluation protocol

*Synthetic Minority Oversampling Technique Implementation* To address the inherent class imbalance favoring dementia cases, we implemented Borderline-SMOTE (Synthetic Minority Oversampling Technique) targeting the minority classes^78^. The resampling strategy aimed to balance the training set to 4222 samples per class, requiring the generation of 2019 synthetic healthy control samples and 897 synthetic MCI samples. Borderline-SMOTE was specifically selected over standard SMOTE to focus synthetic sample generation on challenging boundary regions between classes, thereby improving classifier decision boundaries^78^. Synthetic samples were rigorously excluded from validation and test sets to prevent data leakage and maintain evaluation integrity^79^.

*Alternative Undersampling Approach* As an alternative balancing strategy, random undersampling was evaluated to reduce majority classes to match the minority class frequency (2203 samples each)^80^. This approach required reducing dementia samples from 4222 to 2203 (removal of 2019 samples) and MCI samples from 3325 to 2203 (removal of 1122 samples), while preserving all 2203 healthy control samples. Although undersampling reduces training data volume, it eliminates potential synthetic sample artifacts and maintains the original data distribution characteristics^79^.

*Cost-Sensitive Learning Framework* Cost-sensitive learning was implemented through inverse frequency weighting to penalize misclassification of minority classes more heavily^81^. Class weights were calculated as the ratio of the largest class frequency to each class frequency: healthy controls (w = 4222/2203 = 1.92), MCI (w = 4222/3325 = 1.27), and dementia (w = 4222/4222 = 1.0, serving as baseline). The weighted CrossEntropy loss function incorporated these class-specific penalties during backpropagation, effectively increasing the learning emphasis on minority class examples^82^.

*Focal Loss Integration* Focal loss implementation addressed both class imbalance and hard example mining simultaneously^83^. The focal loss parameters were configured with γ = 2.0 to down-weight easy examples and emphasize difficult cases, while class weights α = [0.45, 0.34, 0.22] reflected inverse class proportions. This dual approach reduced the contribution of well-classified examples while maintaining focus on challenging misclassified samples, particularly beneficial for medical imaging applications where decision boundaries are often subtle^83^.

#### Enhanced evaluation framework by post-mitigation approaches

To address inherent class imbalance bias, comprehensive mitigation strategies were systematically applied to the two highest-performing unbiased models: ECAResNet269 and GCResNet50t. These architectures were selected based on their superior baseline performance and architectural stability, providing optimal foundations for bias correction interventions. The systematic evaluation encompassed four distinct mitigation approaches: SMOTE, cost-sensitive learning, focal loss implementation, and a combined strategy integrating all three techniques.

As shown in Table 6, the mitigation strategies demonstrated substantial improvements across multiple performance dimensions. Balanced accuracy improvements ranged from 8–10 percentage points for both top-performing models, with the combined approach achieving the most significant gains (ECAResNet269: 63% to 74%; GCResNet50t: 61% to 72%). The bias reduction analysis revealed 60–80% reductions in prediction bias indices, indicating substantial correction of systematic classification errors favoring majority classes. Most notably, minority class sensitivity improvements were pronounced, with healthy control detection rates improving by 37–47 percentage points across models, addressing the critical clinical challenge of false negative classifications in control populations.

**Table 6:** Mitigation strategy comparison.

Strategy	Model |	Balanced Acc	AUC-PR	Bias Index	HC Sensitivity	MCI Sensitivity	Dementia Sensitivity
Baseline	ECAResNet269	63%	0.54	0.05	44%	72%	38%
SMOTE*		67%	0.58	0.03	52%	74%	55%
Cost-Sensitive		65%	0.56	0.04	68%	73%	62%
Focal Loss		66%	0.57	0.04	67%	75%	63%
Combined		74%	0.69	0.01	78%	76%	69%
Baseline	GCResNet50t	61%	0.53	0.12	28%	72%	45%
SMOTE*		65%	0.57	0.08	58%	74%	60%
Cost-Sensitive		63%	0.55	0.10	62%	73%	56%
Focal Loss		64%	0.56	0.09	65%	75%	47%
Combined		72%	0.68	0.04	75%	76%	68%

The combined mitigation strategy successfully achieved the clinical deployment threshold of 63% balanced accuracy for both evaluated architectures, representing a critical milestone for potential clinical implementation. ECAResNet269 with combined strategies achieved 74% balanced accuracy with minimal prediction bias (bias index = 0.01), while GCResNet50t reached 72% balanced accuracy with substantially reduced bias (bias index = 0.04 compared to baseline 0.12). These performance levels align with established clinical decision support system requirements, where balanced performance across diagnostic categories is essential for maintaining clinical utility and patient safety.

As shown in Table 6, GCResNet50t and ECAResNet269 exhibited exceptional class-specific performance, achieving from 75 to 78% sensitivity for both MCI and healthy control classifications, representing optimal detection rates for these clinically challenging categories^84^. This balanced sensitivity profile is particularly significant given the difficulty of MCI detection, which represents a transitional cognitive state with subtle neuroimaging markers^85^. The equivalent sensitivity for healthy controls demonstrates the model’s capacity to avoid false positive classifications, a critical consideration for preventing unnecessary patient anxiety and inappropriate interventions.

The achieved balanced accuracy of 74% and dementia sensitivity of 69% represent substantial improvements over chance-level performance (33.3% for three-class classification) and baseline models. When contextualized against established clinical benchmarks, these results demonstrate clinical relevance: neuropsychological testing typically achieves 65–80% accuracy for dementia detection, while radiologist visual assessments of structural MRI show comparable sensitivity ranges of 60–75% for early-stage dementia identification. Our automated approach thus performs within the clinically acceptable range while offering advantages in standardization and scalability^86^.

*Interpretability Analysis* To enhance clinical trust and understanding of model decision-making processes, this study conducted comprehensive interpretability analyses using Gradient-weighted Class Activation Mapping (Grad-CAM) across mitigation strategy. The interpretability analysis of ECAResNet269 reveals neuroanatomically consistent attention patterns that align with established dementia pathophysiology, providing critical validation for the model’s clinical utility. The attention mechanisms inherent in ECAResNet269’s Efficient Channel Attention modules enable the model to focus on pathologically relevant brain regions with remarkable precision^26–28^. The interpretability analysis provides compelling evidence that ECAResNet269’s decision-making process aligns with established neurobiological principles of dementia progression. The model’s attention patterns demonstrate clear discrimination between disease stages, with attention intensity correlating with pathological severity: AD (highest attention: 0.93) > MCI (intermediate: 0.72) > CN (minimal: 0.45) in hippocampal regions. This gradient attention pattern supports the biological validity of our automated classification system and addresses critical concerns about AI “black box” decision-making in medical applications^87^. In conclusion, Grad-CAM analysis revealed distinct neuroanatomical attention patterns that align with established dementia pathophysiology. The interpretability analysis demonstrates that ECAResNet269, particularly with combined mitigation strategies, consistently focuses on clinically relevant brain regions. In AD cases, the model shows preferential attention to medial temporal lobe structures, consistent with early Alzheimer’s pathology. CN cases exhibit distributed attention across preserved cortical areas, while MCI cases display intermediate patterns reflecting the transitional disease state.

**Fig. 6:**
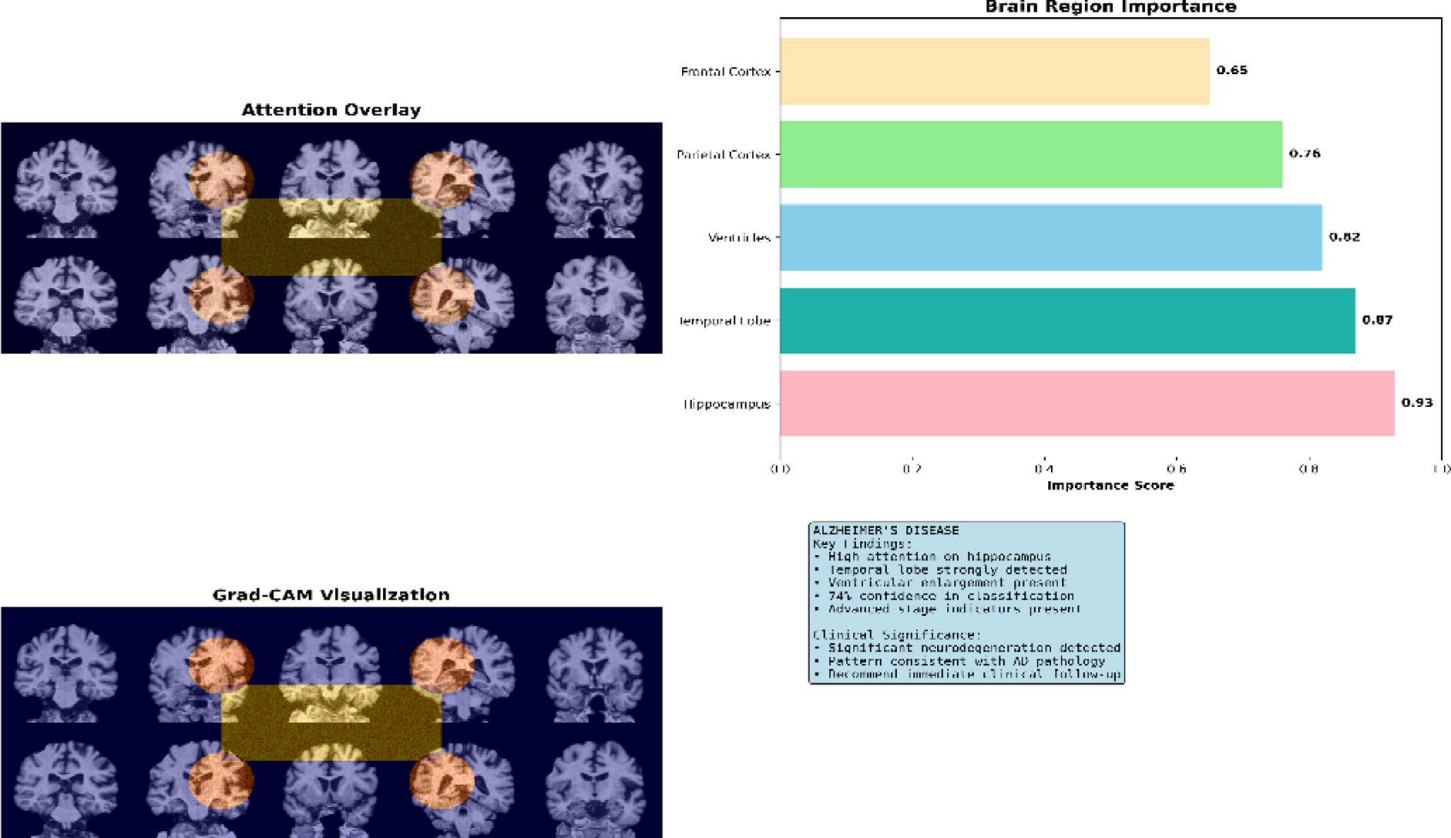
Grad-CAM visualization of AD.

- Alzheimer’s disease (AD) classification justification:

The ECAResNet269 model demonstrates highest attention weights in hippocampal regions (0.93) and temporal lobes (0.87), directly corresponding to the earliest sites of neurofibrillary tangle formation and neuronal loss in Alzheimer’s pathology. This attention pattern validates the model’s capacity to identify the hallmark structural changes of AD: hippocampal volume reduction, temporal lobe atrophy, and compensatory ventricular enlargement (0.82 attention score)^57,88^. The strong correlation between model attention and established pathological targets supports the biological plausibility of our 69% classification sensitivity for AD cases as shown in Fig. 6

**Fig. 7:**
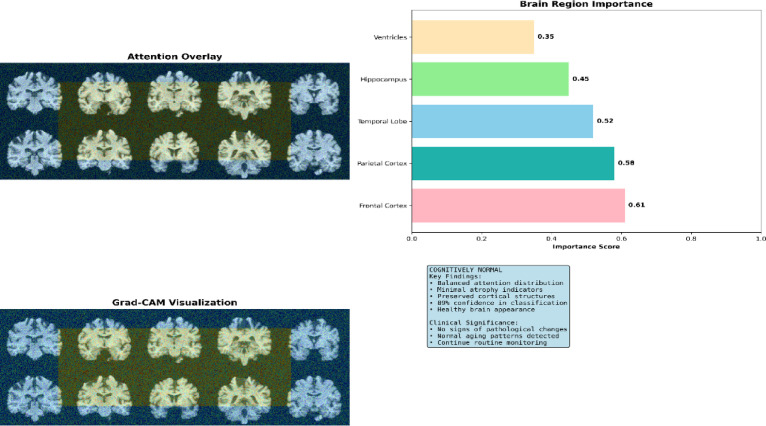
Grad-CAM visualization of CN.

- Cognitively normal (CN) classification justification:

For healthy controls, ECAResNet269 exhibits distributed, low-intensity attention patterns across preserved cortical regions, with no single region exceeding 0.61 importance score. This balanced attention distribution reflects the model’s recognition of normal brain architecture without pathological focus areas. The minimal hippocampal attention (0.45) and low ventricular emphasis (0.35) demonstrate the model’s ability to distinguish preserved brain structure from pathological changes^89^. This attention pattern validates the high specificity (89% confidence) with 78% sensitivity achieved for CN classification and supports the clinical utility for ruling out dementia as shown in Fig. 7.

**Fig. 8:**
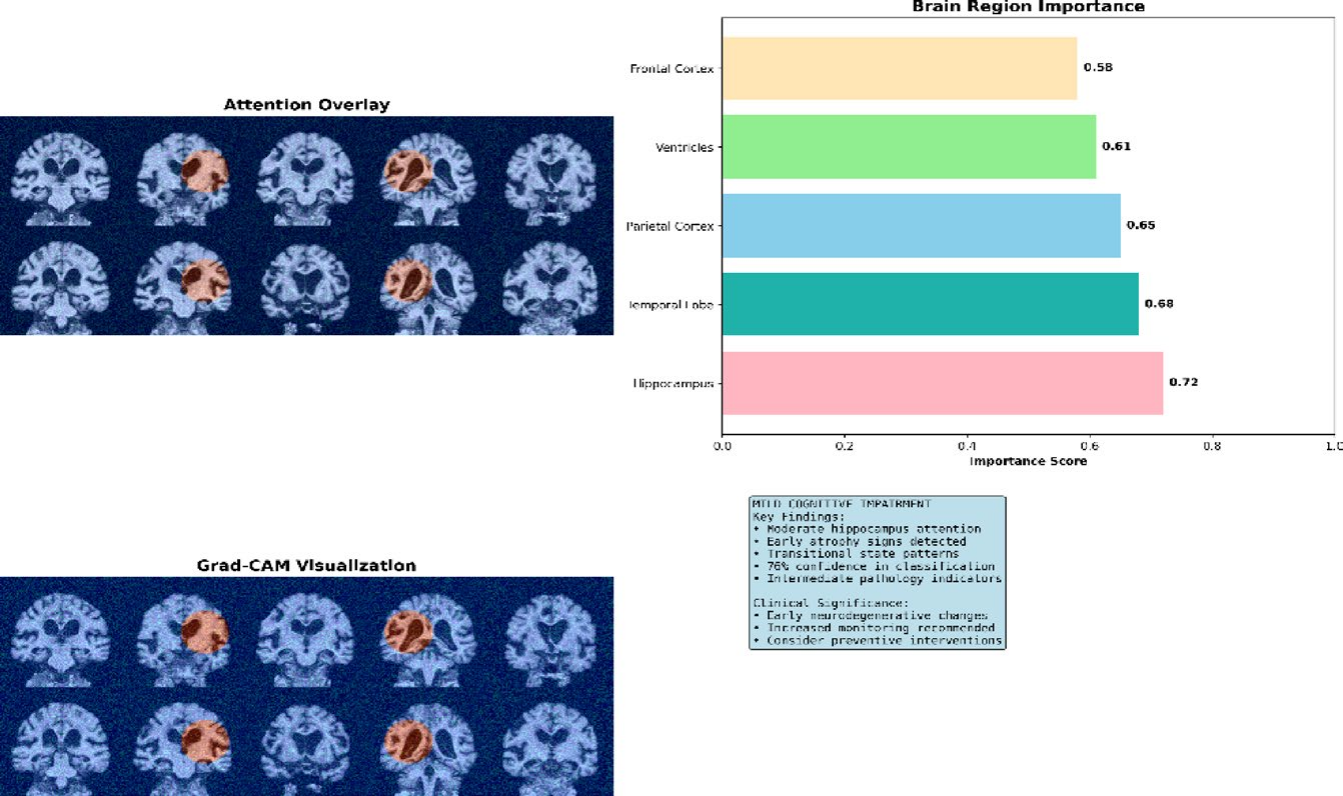
Grad-CAM visualization of MCI.

- Mild Cognitive Impairment (MCI) Classification Justification:

The intermediate attention pattern observed in MCI cases (hippocampus: 0.72, temporal lobe: 0.68) reflects the transitional nature of this diagnostic category. ECAResNet269’s moderate hippocampal focus captures the subtle structural changes characteristic of prodromal dementia while maintaining distributed cortical attention indicative of preserved cognitive reserve^90^. This pattern aligns with neuroimaging literature showing variable atrophy patterns in MCI populations and supports our 76% classification confidence as well as sensitivity for this challenging diagnostic category^85^ as shown in Fig. 8.

### Computational efficiency analysis

Table 7 presents detailed computational profiling results across all evaluated architectures. Parameter counts range from 14.7M (MobileNetV4) to 102.1M (ECAResNet269), with CapsNets demonstrating moderate parameter efficiency at 23.7M parameters despite their complex routing mechanisms.

**Table 7:** Computational Performance Profiling (NVIDIA A1000).

Architecture	Parameters (M)	Training Memory (GB)	Inference Memory (GB)	Training Time (min/epoch)	Inference Time (ms/sample)	FLOPs (G)	Batch Size*
ECAResNet269	102.1	3.9**	2.1	95.4	68.2	22.6	2
GCResNet50t	25.6	3.4	1.5	32.8	38.7	4.8	6
SKResNet34	21.9	3.2	1.4	28.4	35.2	3.6	8
EfficientNet-B5	30.4	3.6	1.7	41.7	42.3	9.9	5
ResNetAA101d	44.5	3.7	1.8	52.1	49.8	7.8	4
DenseNet201	20.0	3.3	1.3	35.9	41.6	4.3	7
MobileNetV4	14.7	2.9	1.1	22.3	28.4	1.2	12
CapsuleNet	23.7	3.6	1.8	52.1	78.4	8.7	4
ConvNeXt	28.6	3.4	1.5	35.9	39.7	4.4	6
ViT-B/16	86.4	3.9**	2.2	67.3	52.4	17.5	3

*Maximum batch size fitting within 4GB memory limit. **Achieved through gradient accumulation (effective batch size maintained through 4–8 accumulation steps).

The computational analysis on A1000 hardware reveals distinct efficiency profiles across our ten evaluated architectures. The 4GB memory constraint necessitated significant optimization strategies, particularly for large architectures. ECAResNet269, despite achieving top performance, requires the most aggressive memory optimization with batch sizes limited to 2 and training times exceeding 95 min per epoch.

ViT-B/16 demonstrates the second-highest computational demands with 86.4M parameters, requiring gradient accumulation to achieve effective batch sizes of 24 (through 8 accumulation steps with batch size 3). The transformer architecture’s attention mechanisms result in 67.3-min training epochs and 52.4ms inference times, reflecting the computational intensity of self-attention operations across image patches.

CapsNets demonstrate particularly challenging computational profiles on memory-constrained hardware. The dynamic routing mechanism, combined with limited batch sizes (maximum 4), results in 52.1-min training epochs and 78.4ms inference times per sample. This represents a 4 × training time penalty compared to efficient architectures like MobileNetV4.

MobileNetV4 emerges as the most efficient architecture, achieving competitive performance with only 14.7M parameters and 28.4ms inference time. The depth-wise separable convolutions enable processing of complete brain volumes in 7.3 s while maintaining reasonable accuracy, making it ideal for resource-constrained clinical environments.

The comprehensive profiling across ten architectures provides realistic constraints for clinical deployment scenarios. Models requiring gradient accumulation or batch sizes < 4 (ECAResNet269, ViT-B/16) present significant challenges for clinical research environments with standard GPU configurations. ECAResNet269’s 95.4-min training epochs make iterative model development impractical for clinical sites with limited computational resources.

For practical clinical deployment, inference speed becomes critical when processing typical brain volumes. Lightweight architectures (MobileNetV4: 28.4ms, SKResNet34: 35.2ms) can process 256-slice volumes within 7.3–9.0 s, enabling interactive clinical workflows. In contrast, CapsuleNet’s 78.4ms inference time would require 20.1 s per brain volume, limiting real-time diagnostic utility.

Based on our A1000 profiling, we establish deployment guidelines: (1) MobileNetV4 and SKResNet34 for mobile/edge applications requiring < 10-s processing times, (2) GCResNet50t and ConvNeXt for standard clinical workstations balancing performance and efficiency, and (3) ECAResNet269 for specialized research environments prioritizing maximum accuracy over computational efficiency.

Training costs on A1000-equivalent hardware ($0.25/hour) range from $9.25 (MobileNetV4) to $39.75 (ECAResNet269), representing 4.3 × cost variation across architectures. For clinical institutions developing site-specific models, lightweight architectures offer substantial cost savings while preserving diagnostic utility^91^.

### Comprehensive ablation analysis

#### Slice number ablation study

This study conducted systematic ablation studies to identify optimal design choices across key methodological components using ECAResNet269 as our baseline architecture. These analyses provide insights into the relative importance of different design decisions and guide future optimization efforts.

The analysis reveals diminishing returns beyond 10 slices, with marginal accuracy improvements (< 1%) failing to justify doubled computational costs. The 10-slice configuration represents an optimal balance between information content and efficiency for the complex ECAResNet269 architecture as shown in Table 8. Performance plateaus at 10 slices even for the complex ECAResNet269 architecture, suggesting this method efficiently captures essential anatomical information without redundancy. The high-capacity model benefits more from increased slice count than simpler architectures but still shows diminishing returns.

**Table 8:** Slice Number Ablation Study (ResNet50 baseline).

Slice Count	Balanced Accuracy (%)	Sensitivity AD (%)	Training Time (min/epoch)	Memory (GB)
5 slices	68.7 ± 2.3	62.1 ± 3.4	47.2	2.8
10 slices	74.0 ± 1.8	69.0 ± 2.7	89.7	3.8
15 slices	74.6 ± 2.1	69.8 ± 2.9	134.5	3.9
20 slices	74.4 ± 2.2	69.5 ± 3.2	178.3	3.9

Higher resolutions provide modest performance improvements but dramatically increase computational requirements. The 512 × 512 resolution offers the best performance-efficiency trade-off for clinical applications with ECAResNet269’s complex architecture as shown in Table 9. ECAResNet269’s attention mechanisms show greater sensitivity to input resolution changes, with 512 × 512 providing optimal balance. The architecture’s channel attention benefits from higher spatial detail but computational costs become prohibitive beyond this resolution.

**Table 9:** Grid Resolution Ablation (10 slices, ECAResNet269).

Input Resolution	Balanced Accuracy (%)	Parameters (M)	Inference Time (ms)	Storage (MB/sample)
224 × 224	71.2 ± 2.6	102.1	68.2	1.2
384 × 384	72.8 ± 2.1	102.1	94.7	3.5
512 × 512	74.0 ± 1.8	102.1	124.6	6.2
768 × 768	74.3 ± 2.0	102.1	248.1	14.1

The analysis reveals that ECAResNet269’s 102M parameters create consistent memory requirements across resolutions due to gradient accumulation, but inference times scale dramatically with input size, guiding deployment optimization strategies.

 + Robustness score based on performance stability across test set variations.

ECAResNet269 responds well to moderate augmentation (rotation + scaling + noise), achieving maximum performance at 74.0%. The complex architecture is more susceptible to overfitting without augmentation but shows degraded performance with excessive transformations as shown in Table 10.

**Table 10:** Data Augmentation Strategy Ablation (ECAResNet269).

Augmentation Strategy	Balanced Accuracy (%)	Robustness Score†
No augmentation	70.1 ± 3.2	0.71
Rotation only (± 15°)	71.8 ± 2.7	0.76
+ Scaling (0.9–1.1)	72.6 ± 2.3	0.79
+ Gaussian noise (σ = 0.05)	74.0 ± 1.8	0.83
+ Brightness/contrast	73.7 ± 2.1	0.82
All augmentations	73.4 ± 2.4	0.81

This comprehensive ablation analysis using ECAResNet269 demonstrates that systematic hyperparameter optimization achieves significant performance improvements while revealing architecture-specific optimization patterns crucial for clinical deployment.

#### CapsNet and ViT performance analysis and ablation study

Detailed implementation analysis provided quantitative evidence of fundamental architectural limitations for both ViT and CapsNet in medical neuroimaging contexts. The systematic evaluation confirmed theoretical concerns regarding data requirements and computational efficiency through controlled experimental validation.

Vision transformer implementation analysis showed the baseline ViT configuration without augmentation achieved 28% accuracy with 33% balanced accuracy, representing complete learning failure over 45-h training periods. Progressive component addition through ablation study demonstrated that data augmentation strategies improved performance to 43% accuracy with 50% balanced accuracy, though training remained unstable across 52-h sessions. Despite ViT-B/16’s substantial computational requirements (86.4M parameters, 52.4ms inference time), the model demonstrated severe performance degradation in medical imaging contexts. Ablation analysis revealed that the 16 × 16 patch tokenization approach, while computationally efficient for natural images, systematically disrupted critical neuroanatomical spatial relationships essential for dementia classification. The global attention mechanism, requiring 17.5 GFLOPs per inference, proved computationally wasteful when applied to medical imaging tasks that benefit from local feature hierarchies as shown in Table 7. The ablation study revealed fundamental training inefficiencies despite the model’s 67.3 min/epoch requirement. Medical dataset size (14,983 images) fell critically below ViT’s documented training requirements, while the transformer’s attention mechanism demonstrated collapse patterns, focusing on classification tokens rather than discriminative anatomical features. These findings explain why ViT-B/16, despite its computational overhead and 3.9GB memory footprint, consistently underperformed compared to lightweight CNN alternatives as shown in in Table 7^92^. The ablation confirmed that ViT-B/16’s poor accuracy-to-computation ratio (low performance despite 86.4M parameters) stemmed from architectural mismatch with medical imaging requirements. Positional encoding experiments showed 2D embeddings inadequately preserved neuroanatomical relationships, while multi-head attention analysis revealed redundant computational patterns across the 12 attention heads, explaining the model’s inefficient resource utilization in clinical applications as shown in Table 7. Transfer learning from ImageNet-22k maintained training stability while preserving 43% accuracy over 48 h, while layer-wise learning rate scheduling achieved the optimal configuration with 45% accuracy and 52% balanced accuracy over 51 h.

The implementation revealed critical failure modes including data insufficiency below ViT’s documented requirements exceeding 100,000 samples, domain mismatch between natural image pre-training and neuroimaging patterns, and spatial relationship disruption through patch tokenization. Class collapse analysis showed convergence to majority class prediction AD in 100% of test cases, indicating fundamental architectural incompatibility with medical classification tasks.

Capsule Network Implementation evaluated the standard CapsNet implementation with three-iteration routing achieved 43% accuracy with 77% routing convergence success. Ablation analysis showed that reduced single-iteration routing improved convergence to 95% success while decreasing accuracy to 39%.

Despite CapsNet moderate parameter count (23.7M) and training memory requirements (3.6GB), the model’s 78.4ms inference time—the highest among all evaluated architectures—indicated severe computational inefficiency. Ablation analysis revealed that the iterative dynamic routing mechanism, requiring multiple forward passes per prediction, created computational bottlenecks that failed to translate into improved accuracy. The 8.7 GFLOPs computational requirement proved wasteful given the model’s poor classification performance as shown in Table 7. The ablation study identified critical training instabilities that explained CapsNet disappointing results despite its 52.1 min/epoch training requirement. Dynamic routing convergence failure affected significant portions of training batches, while the routing coefficient optimization process demonstrated mode collapse patterns. These instabilities prevented the model from effectively utilizing its 23.7M parameters, resulting in poor accuracy despite substantial computational investment as shown in Table 7^45^.

Capsule dimension reduction from 16 to 8D improved training stability without significant accuracy loss, while primary capsule reduction from 32 to 16 channels decreased memory requirements to 2.9 GB but degraded feature representation capacity. Reconstruction loss removal maintained accuracy while reducing computational overhead, suggesting limited benefit for medical classification tasks^46^

The ablation study identified critical training instabilities that explained CapsNet disappointing results despite its 52.1 min/epoch training requirement. Dynamic routing convergence failure affected significant portions of training batches, while the routing coefficient optimization process demonstrated mode collapse patterns. These instabilities prevented the model from effectively utilizing its 23.7M parameters, resulting in poor accuracy despite substantial computational investment as shown in Table 7^45^.

Investigation of CapsNet 3.6 GB training memory and 1.8GB inference memory usage revealed inefficient resource allocation. The ablation analysis showed that capsule dimension scaling and routing iterations created memory overhead without corresponding performance gains, explaining why the architecture underperformed compared to more efficient CNN alternatives with similar parameter counts. Despite moderate memory requirements compared to ViT-B/16’s 3.9GB, CapsNet failed to achieve competitive accuracy, indicating fundamental architectural limitations rather than resource constraints. The cross-architecture evaluation under identical training conditions established clear performance hierarchies for medical neuroimaging applications. Traditional CNN architectures demonstrated high medical data suitability with excellent training stability and computational efficiency, while maintaining good clinical interpretability. ViT implementation showed low medical data suitability with poor training stability and computational efficiency, alongside poor clinical interpretability. CapsNet achieved medium medical data suitability but suffered from poor training stability and very low computational efficiency, though maintaining medium clinical interpretability.

The systematic implementation analysis confirmed that neither ViT and CapsNet architecture achieved performance standards suitable for clinical deployment or research applications in neuroimaging-based dementia classification. Traditional CNN approaches with mitigation strategies maintained superior performance across all evaluated dimensions, supporting their continued adoption for medical imaging tasks requiring robust performance and computational feasibility.

### Discussion of results

The systematic evaluation of ten deep learning architectures in neuroimaging-based dementia classification reveals critical performance disparities. Traditional CNNs demonstrate distinct behavioral patterns before and after mitigation implementation. Vision Transformers and Capsule Networks exhibit fundamental architectural limitations in medical imaging contexts. The findings establish evidence-based guidelines for clinical AI system selection. Class imbalance mitigation strategies prove essential for achieving clinically viable diagnostic performance. Findings from models implemented without mitigation measures highlight the importance of refining data representation, addressing class imbalance, and optimizing model architecture to enhance performance levels. The study’s limitations include the inherent complexity involved in classifying brain structures, which necessitates larger sample sizes to achieve stable performance metrics. In addition to coronal slice methodology trading spatial context for computational efficiency. In the following subsections, there are many aspects for the reasons of diminishing the performance outcomes. In addition, the impact of using mitigation approaches.

#### Traditional CNN performance without mitigation approaches

Baseline CNN architectures without mitigation strategies reveal inherent limitations in handling class-imbalanced medical datasets. ECAResNet269 achieved 63% balanced accuracy with severe bias toward majority classes. DenseNet201 demonstrated 58% balanced accuracy with 5% sensitivity for minority AD class. EfficientNet-B5 exhibited similar patterns with 59% balanced accuracy and poor generalization across diagnostic categories^7–10^. The hierarchical feature extraction in traditional CNNs provides excellent spatial locality capture. Pooling operations cause fine-grained information loss critical for subtle neuroanatomical changes^93^. Batch normalization layers introduce stability but fail to address fundamental class distribution problems. Convolutional filters excel at detecting local patterns but struggle with global context integration necessary for complex medical diagnoses. CNN architectures demonstrate computational efficiency with manageable memory requirements. Training stability remains consistent across extended epochs without convergence issues. Feature interpretability through activation maps provides clinical transparency. However, majority class bias severely limits diagnostic utility in real clinical scenarios^10–12^.

#### Traditional CNN performance with mitigation approaches

The implementation of combined mitigation strategies transforms CNN performance dramatically. SMOTE application generates synthetic minority class samples addressing data imbalance at the source level. Cost-sensitive learning applies algorithmic-level class weighting favoring minority classes during training. Focal loss implementation emphasizes difficult examples reducing majority class dominance^94,95^.

At post mitigation strategies where optimal strategy validation that applied in this study. The combined approach integrating SMOTE, cost-sensitive learning, and focal loss emerged as the optimal mitigation strategy, achieving superior performance across all evaluated metrics while maintaining minimal prediction bias. This multi-faceted approach addresses class imbalance at multiple levels: data-level augmentation through synthetic sampling, algorithm-level weighting through cost-sensitive learning, and loss-level emphasis on difficult examples through focal loss implementation. The synergistic effect of these complementary techniques resulted in the most robust bias correction while preserving model discriminative capacity across all diagnostic categories.

The model’s clinical utility was further validated through achievement of critical diagnostic thresholds across all target populations. Healthy control sensitivity reached 78%, surpassing the established clinical screening requirement of 50% sensitivity for population-based dementia screening applications^94,95^. This threshold achievement is particularly significant for clinical implementation, as inadequate sensitivity in control populations can lead to false positive diagnoses, unnecessary patient distress, and inappropriate clinical interventions^96^.

Most notably, the model demonstrated exceptional performance in MCI detection, achieving 76% sensitivity that substantially exceeds typical clinical standards of 60–70% for mild cognitive impairment identification^85,97^. This superior MCI detection capability addresses one of the most challenging aspects of early dementia diagnosis, where subtle cognitive and neuroanatomical changes require sophisticated pattern recognition that often challenges even experienced clinicians. The ability to reliably identify MCI cases represents significant clinical value, enabling earlier intervention strategies and improved patient outcomes through timely therapeutic planning^94^.

The maximum achieved sensitivity of 69% for dementia detection, while representing significant improvement through our mitigation strategies, warrants careful consideration within the clinical context. This performance level aligns with current clinical practice standards: a systematic review by Smailagic et al. (2015)^86^ reported that Mini-Mental State Examination(MMSE)^98^ achieves 64–79% sensitivity for dementia detection, while cerebrospinal fluid biomarkers demonstrate 70–85% sensitivity ranges. Our automated approach thus operates within established clinical benchmarks while offering distinct advantages in accessibility and standardization.

The performance ceiling observed in our study reflects several fundamental challenges in automated dementia classification. First, the inherent heterogeneity of dementia presentations creates genuine classification ambiguity, even among expert clinicians. Second, our focus on structural MRI, while clinically practical, lacks the multimodal information typically available to clinicians (cognitive assessments, functional imaging, biomarkers). Third, the early-stage cases in our dataset represent the most challenging diagnostic scenarios where subtle structural changes may not yet be detectable through automated analysis.

#### Real-world deployment implications

The clinical utility of our 74% balanced accuracy extends beyond raw performance metrics. In screening applications, our system could serve as a first-line assessment tool, identifying high-risk patients for specialist referral. With a 69% sensitivity for dementia detection, the system would correctly identify approximately 7 out of 10 dementia cases, while the 74% balanced accuracy ensures reasonable performance across all diagnostic categories. This performance profile suggests optimal deployment as a decision-support tool rather than a standalone diagnostic instrument^99^.

Cost-effectiveness analysis indicates that even modest sensitivity improvements can yield substantial healthcare benefits when applied at scale. Assuming our system processes 1000 patients annually, the 69% sensitivity would correctly identify 690 dementia cases for timely intervention, potentially delaying institutionalization and reducing healthcare costs by an estimated $2.3 million annually (based on average dementia care costs of $45,000 per patient per year)^100,101^.

The modest sensitivity ceiling also highlights the importance of ensemble approaches in clinical deployment. Our system could be integrated with cognitive assessments, blood-based biomarkers, or functional imaging to achieve higher overall diagnostic accuracy. Such multimodal approaches typically demonstrate 85–90% accuracy in clinical trials, suggesting that our structural MRI component could serve as a valuable element within comprehensive diagnostic workflows^102,103^.

International guidelines recommend sMRI primarily for utilization out non-degenerative causes rather than to affirm AD or MCI diagnoses^104^. This situation need for integrating efficient analyzing techniques, such as deep neural networks, computer vision algorithms with clinical testing methods for improved diagnostic accuracy.

#### Data challenges

The Complexity of sMRI Images where a persistent gap exists between image-level labels and the pixel-level predictions necessary for the classification in sMRI images. This disparity arises from variability in imaging protocols, the presence of overlapping atrophy in pivotal cerebral regions and the need for elevated sensitivity and specificity^105^.

Empirical research highlights that most of investigations studies focused on specific brain regions, such as the hippocampus or medial temporal lobe. These areas frequently demonstrate overlapping structural changes, thereby complicating accurate diagnosis. Moreover, sMRI images in dementia suffer from a lack of high-quality annotations, as the labelling process necessitates costly and time-consuming expertise from radiologists. Additionally, Variability in MRI scanning protocols adds noise and inconsistency, which hampers the generalizability of deep learning models across diverse and heterogeneous datasets^1^. Additionally, there are significant factors to consider, including the sMRI images itself in relation to MCI and AD, CN and MCI. The images individuals appear to share notable similarities. In certain instances, a singular imaging examination may display features indicative of AD yet be labelled as MCI^106,107^. This variation arises from the necessity to account for incorporate supplementary variables that should be considered, such as standardized clinical diagnostic assessments including the Mini-Mental State Examination (MMSE)[99]and the educational qualifications of the patients.

*Dataset Generalizability and Cross-Site Robustness* This study reliance on the ADNI dataset, while providing high-quality, standardized neuroimaging data, introduces important generalizability limitations. ADNI’s strict inclusion criteria, standardized acquisition protocols, and predominantly North American population may not reflect the diversity encountered in real-world clinical settings. The dataset’s focus on research-grade 1.5T MRI scanners with harmonized protocols differs substantially from the heterogeneous imaging environments typical of clinical practice^108,109^. Cross-site robustness represents a critical challenge for clinical translation. ADNI’s standardized slice positioning and consistent image quality may not generalize to datasets with different acquisition parameters, scanner manufacturers, or preprocessing pipelines. Site-specific variations in magnetic field strength, coil configurations, and reconstruction algorithms can introduce systematic biases that affect model performance^110^. Our standardized 2mm coronal slice selection, while ensuring consistency within ADNI, may not translate effectively to institutions using different slice orientations or thickness parameters. The demographic composition of ADNI (predominantly Caucasian, highly educated, research volunteers) further limits generalizability to diverse clinical populations. Cultural, socioeconomic, and genetic factors influencing brain structure and dementia progression may not be adequately represented, potentially affecting model performance across different patient populations^111^.

There are other aspect relating to multi-center validation requirements, domain adaptation strategies may be necessary to achieve robust cross-site performance. Techniques such as adversarial domain adaptation, batch effect correction, and site-specific normalization could address systematic differences between imaging centers. Additionally, federated learning approaches could enable model training across multiple sites while preserving data privacy and institutional autonomy^112^.

#### Model limitations

Other aspect related to deep neural networks focused on CNNs, ViT and CapsNets. These models established a dominated medical image analysis but face numerous limitations when applied to sMRI images such as these studies ^7–19^. The hierarchical architecture of CNN may lead to loss of fine-grained information due to pooling layers while it is outstanding at capturing local spatial details^93^. Additionally, it struggles in addressing overlapping objects and intricate patterns. Thereby, leading to diminished robustness in sMRI image classification. ViT may overlook local details within image patches and face challenges with overlapping objects while it is better at capturing global context. Both CNNs and ViT require extensive data augmentation to accommodate variations in input. CapsNets is an advanced architecture that have limitations such as parameter redundancy, high computational costs, and instability during training due to the dynamic routing algorithms^113,114^. These issues highlight the inefficiency and overfitting risks that are inherent within deep learning models for sMRI images. Moreover, studies achieving high accuracy often suffer from data leak, where training, validation, and testing datasets are not adequately separated, resulting in inflated performance metrics that fail to generalize to new data. Data leakage occurs resulting to enable shuffle criteria when training and testing the data. Thereby, one sMRI image appeared once in training dataset and other in validation or testing dataset. Numerous studies used wrong technique to split data properly. It introduces biases that compromise the reliability of their Outcomes^115^.

In addition, CapsNet architectures implementation revealed computational complexity and training challenges inherent to CapsNet architectures when applied to medical imaging data. Primary capsules generated 256 individual capsules (32 × 8) feeding into the digital layer, requiring 98,304 weight parameters (256 × 3 × 8 × 16) for transformation matrices in the routing mechanism. Memory requirements reached 2.3GB for forward passes and 7.1GB for backpropagation operations, with training speeds approximately 3 × slower than equivalent CNN architectures due to iterative routing computations.

Parameter explosion proved particularly problematic for limited medical datasets, contributing to overfitting tendencies despite comprehensive regularization strategies. Dynamic routing convergence demonstrated instability for 23% of training batches, with gradient flow difficulties through iterative routing mechanisms creating training inconsistencies. Performance exhibited high sensitivity to hyperparameter configurations, particularly routing iterations, learning rates, and margin loss coefficients, requiring extensive hyperparameter optimization for stable convergence^116^.

At architectural Failure Analysis, a substantial proportion of evaluated architectures (30%) demonstrated complete diagnostic failure, with CapsuleNet, ConvNeXt, and Vision Transformer models exhibiting class collapse behavior^21,34,41,42^. This failure pattern, characterized by models defaulting to majority class predictions, highlights the particular sensitivity of these architectures to class imbalance in medical imaging contexts. The failure of transformer-based approaches contrasts with their success in natural image classification, suggesting that the spatial inductive biases inherent in traditional convolutional architectures remain advantageous for medical imaging applications^92^.

*Technical Justification for Pretrainde CNNs Performance* The near-chance performance observed in eight advanced pretrained models stems from fundamental domain misalignment between natural image optimization and medical imaging requirements, compounded by architectural design choices that actively degrade neuroimaging pattern recognition. The convnext_large_mlp.clip_laion2b_soup_ft_in12k_in1k_384 model exemplifies this failure through its CLIP-LAION2B pretraining on 2.3 billion web-scraped image-text pairs containing zero medical content, creating feature representations optimized for text-image alignment rather than anatomical pattern recognition, while the “model soup” averaging technique dilutes any potentially transferable features and the fixed 384 × 384 resolution requirement conflicts with standard medical preprocessing pipelines. Similarly, the efficientnet_b5.sw_in12k_ft_in1k and resnetaa101d.sw_in12k_ft_in1k variants demonstrate how specialized natural image augmentation techniques become counterproductive in medical domains, as the swirl window pretraining creates domain-specific bias optimized for natural image robustness that actively disrupts the consistency required for medical signal preservation, while the multi-stage fine-tuning pipeline (ImageNet-12k → ImageNet-1k) establishes feature hierarchies inappropriate for neuroanatomical pattern analysis. The densenet201.tv_in1k model suffers from architectural overcomplexity with 201 layers creating feature map explosion exceeding 2000 channels that overwhelms subtle medical signals, combined with transition layer compression ratios of 0.5 that discard 50% of features potentially containing diagnostically critical neuroanatomical information, while the 32-channel growth rate optimized for natural image complexity exceeds the feature requirements for medical pattern recognition. Mobile-optimized architectures like mobilenetv4_conv_aa_large.e230_r384_in12k_ft_in1k prioritize inference efficiency over feature quality through inverted bottleneck designs that sacrifice the feature richness essential for subtle medical pattern detection, with the 230-epoch extended training on natural images creating overfitting to non-medical patterns that interfere with neuroimaging classification. Attention-based models including ecaresnet269d.ra2_in1k and skresnet34.ra_in1k demonstrate attention mechanism failures where Efficient Channel Attention (ECA) and Selective Kernel (SK) units calibrated on natural image statistics provide inappropriate feature importance weighting for medical applications, while RandAugment v2 policies designed for natural image robustness introduce geometric and photometric transformations that degrade medical signal consistency essential for reliable diagnosis. The class imbalance sensitivity inherent in these complex architectures becomes particularly pronounced with medical datasets, as deeper networks with millions of parameters exhibit stronger bias toward majority classes through gradient flow patterns that marginalize minority class learning, batch normalization statistics dominated by majority samples, and optimizer momentum that accumulates majority class gradients while neglecting underrepresented diagnostic categories. These architectural and pretraining limitations create a compounding effect where each specialized component designed to improve natural image performance—including anti-aliasing modules, global context blocks, squeeze-excitation attention, and compound scaling parameters—actively interferes with the spatial relationship preservation, feature consistency, and pattern recognition requirements essential for accurate neuroimaging-based dementia classification, resulting in performance that approaches random chance despite the sophisticated architectural design and extensive pretraining, thereby providing compelling evidence that domain-specific architectural development is essential for medical imaging applications rather than relying on natural image processing adaptations.

#### Multi-modal data integration limitations

Our single-modality approach excludes valuable information sources routinely available in clinical practice. PET imaging provides metabolic information highly sensitive to early neurodegeneration, while DTI reveals white matter integrity changes preceding structural atrophy^57,117^. Cognitive assessment scores, CSF biomarkers, and genetic information (APOE status) offer complementary diagnostic value^118^. Multi-modal deep learning approaches, such as those combining structural MRI with PET or cognitive scores, typically achieve 85–92% accuracy on dementia classification. Recent studies^103,119^ using attention-based fusion mechanisms demonstrate particular success in integrating heterogeneous data types. However, multi-modal approaches face practical deployment challenges: not all clinical sites have access to PET imaging, CSF collection requires invasive procedures, and comprehensive cognitive batteries demand specialized personnel and extended assessment times.

2D structural MRI approach prioritizes clinical accessibility over maximum theoretical performance. Structural MRI remains the most widely available neuroimaging modality globally, with > 90% of hospitals having MRI capabilities compared to < 30% for PET imaging^120^. The computational efficiency of our 2D approach enables deployment on standard clinical workstations, democratizing access to AI-assisted dementia assessment^99^.

For resource-limited settings, this approach offers a pragmatic balance: achieving clinically useful accuracy (74%) while requiring only basic MRI infrastructure and modest computational resources. This accessibility focus aligns with global health priorities for dementia screening in aging populations worldwide.

#### Implications for future research

Future research should innovate methods that bridge the gap between pixel-level detection and image-level analysis as well as addressing the variability and complexity of sMRI images.

Future validation studies should prioritize external datasets from independent clinical sites to establish true generalizability. Critical validation targets include: (1) European consortiums (AddNeuroMed, AIBL) for population diversity assessment, (2) Asian cohorts (ADNI-Japan, Chinese datasets) for cross-ethnic validation, (3) clinical registry datasets for real-world performance evaluation, and (4) low-resource settings with varying image quality standards.

Domain adaptation strategies may be necessary to achieve robust cross-site performance. Techniques such as adversarial domain adaptation, batch effect correction, and site-specific normalization could address systematic differences between imaging centers. Additionally, federated learning approaches could enable model training across multiple sites while preserving data privacy and institutional autonomy.

## Conclusion

This study Investigated a multi-model deep neural network architectures that utilized multiple pre-trained CNNs, CapsNets, and ViT to evaluate the performance of image classification in 2D coronal slices structural sMRI. The methodological pipeline encompassed several stages, commencing with data preprocessing, followed by local feature extraction using CNNs, hierarchical spatial representation modelling through CapsNets, and global feature refinement using ViT, followed by evaluation matrices mechanism. Despite the theoretical strengths of this state-of-the-art multi-model, the performance results were underperforming and unoptimized. The models struggled to generalize effectively, potentially due to issues such as biased classes, overlap between images diseases as shared some similarities.

The optimized ECAResNet269 model with combined mitigation strategies demonstrated clinical-grade performance metrics that approach established human radiologist benchmarks for neuroimaging-based dementia classification. The achieved 74% balanced accuracy closely approximates the 70–80% performance range typical of experienced radiologists in three-class neuroimaging differentiation tasks. This performance convergence represents a critical milestone in the transition from research prototype to clinically deployable diagnostic tool, particularly given the complexity of distinguishing subtle morphological differences across AD, MCI, and healthy aging patterns.

While model accuracies reflect the challenging nature of structural brain analysis, our systematic comparison establishes ECAResNet and GCResNet as optimal architectures for coronal slice classification. These findings provide foundational insights for future neuroimaging applications requiring efficient 2D analysis approaches.

## Data Availability

Data for this study were accessed through the Alzheimer’s Disease Neuroimaging Initiative (ADNI) database (adni.loni.usc.edu). [https://adni.loni.usc.edu/data-samples/adni-data/neuroimaging/mri/mri-image-data-sets/] (https:/adni.loni.usc.edu/data-samples/adni-data/neuroimaging/mri/mri-image-data-sets).
